# Biofabrication for spatial control of multiscale biological crosstalk in tissue models

**DOI:** 10.1038/s44385-026-00102-9

**Published:** 2026-08-20

**Authors:** Goeun Yoon, Jihwan Kim, Jinah Jang

**Affiliations:** 1 Department of Mechanical Engineering, Pohang University of Science and Technology (POSTECH), Pohang, Republic of Korea; 2 Center for 3D Organ Printing and Stem Cells, Pohang University of Science and Technology (POSTECH), Pohang, Republic of Korea

**Keywords:** Biological techniques, Biophysics, Biotechnology, Cell biology, Computational biology and bioinformatics, Engineering, Systems biology

## Abstract

Native tissue crosstalk depends on spatial organization, temporal dynamics, and mechanical context, yet 2D cultures and stochastic organoids provide limited control over multicellular and matrix interfaces. This review categorizes biological crosstalk into intercellular, cell–extracellular, and intertissue communication and surveys biofabrication strategies that reconstruct these interactions. We propose a framework for designing physiologically relevant tissue models for mechanistic disease studies and therapeutic discovery.

## Introduction

Understanding the behavior of cells and tissues and applying that knowledge to identify underlying pathophysiology can lead to effective treatments for numerous diseases. To minimize experimental variability, cells are isolated and cultured in vitro, where cellular behavior and metabolism are observed and modified using candidate therapeutics (e.g., pharmacological drugs, bioactive compounds, and biologics). These cells are conventionally cultured as two-dimensional (2D) monolayers; however, cell behavior is not determined solely by intrinsic cellular properties but spatially and temporally encoded through complex biological crosstalk. 2D systems lack essential features of these types of communication, which limits the translational relevance of findings derived from purely planar cultures. Therefore, cells are increasingly cultured in 3D to modify their 3D architecture, extracellular matrix (ECM) mechanics, and multi-tissue organization. In one widely adopted technique, organoid culture, cells self-organize into 3D structures that reproduce key aspects of an organ’s cellular composition, organization, and function^1^. Unlike 2D monolayers, organoids support 3D cell–cell contacts and contact-dependent morphogenesis^2^, thus providing a context in which spatiotemporal cellular interactions emerge within a mechanically and geometrically relevant 3D environment^3,4^.

While organoids offer advantages in recreating cell–cell contact, they remain fundamentally limited in recapitulating geometrical cues by distant signaling, as organoid morphogenesis is primarily driven by stochastic self-organization. The variability and difficulty in tissue positioning and boundary formation hinder the formation of a larger tissue-scale architecture^5^, as soluble factor gradients typically arise passively through diffusion within the organoids^6^. Therefore, current organoids are often limited to several hundred micrometer-scale spherical structures and struggle to recapitulate anisotropic and macroscale tissue architectures. In addition, organoids are commonly generated without a defined external ECM, which limits systematic and orthogonal control over biochemical composition and mechanical properties. In the early stage of tissue formation, integrin signaling with the surrounding ECM enhances adhesion and protects from anoikis. Moreover, the ECM’s stiffness affects cell fate (i.e., neural, osteogenic, or adipogenic lineages), supporting the importance of ECM modification to achieve intended cell differentiation. Finally, organoids often fail to reconstruct the organized multi-tissue interfaces, perfusable vasculature, and stromal or immune compartments required to interrogate intertissue crosstalk^7^. The paracrine and endocrine signaling delivered through the integration of multiple tissues affects long-term survival and function maintenance, which is otherwise challenging to mimic in a dynamic environment. Collectively, these limitations reduce reproducibility and make communication largely an emergent outcome rather than an experimentally controllable variable. Mechanistic inference, therefore, motivates platforms that can impose deterministic spatiotemporal organization and defined ECM boundary conditions.

When integrated with organoid systems, biofabrication-based strategies—including micromolding, bioprinting, and bioassembly—provide a complementary approach to achieve deterministic control over spatiotemporal intercellular interactions within engineered tissues^8^. Specifically, biofabrication enables morphological regulation of cellular arrangements with defined intercellular distances, angles, and sequences. This in turn allows the formation of defined tissue geometries such as reproducible organoids, engineered epithelial architectures, and perfusable vessel structures across multiple length scales beyond diffusion-limited constraints. In parallel, ECM-mediated regulation precisely modulates cell–ECM interactions through the control of biochemical composition, mechanical stiffness, and anisotropic architecture as well as the application of external mechanical cues. These capabilities provide a well-defined microenvironment that governs cell fate, mechanotransduction, and functional maturation. Furthermore, by controlling the assembly of multiple organoids or spatially defined heterogeneous tissue architectures, biofabrication enables precise regulation of intertissue communication. This allows the reconstruction of complex tissue interfaces such as tumor–stroma boundaries and vascularized organoid systems with defined intertissue distances and directional signaling. Collectively, these approaches transform cell–cell and cell–ECM interactions from passive consequences of self-organization into controllable and quantifiable parameters^1^.

**Fig. 1:**
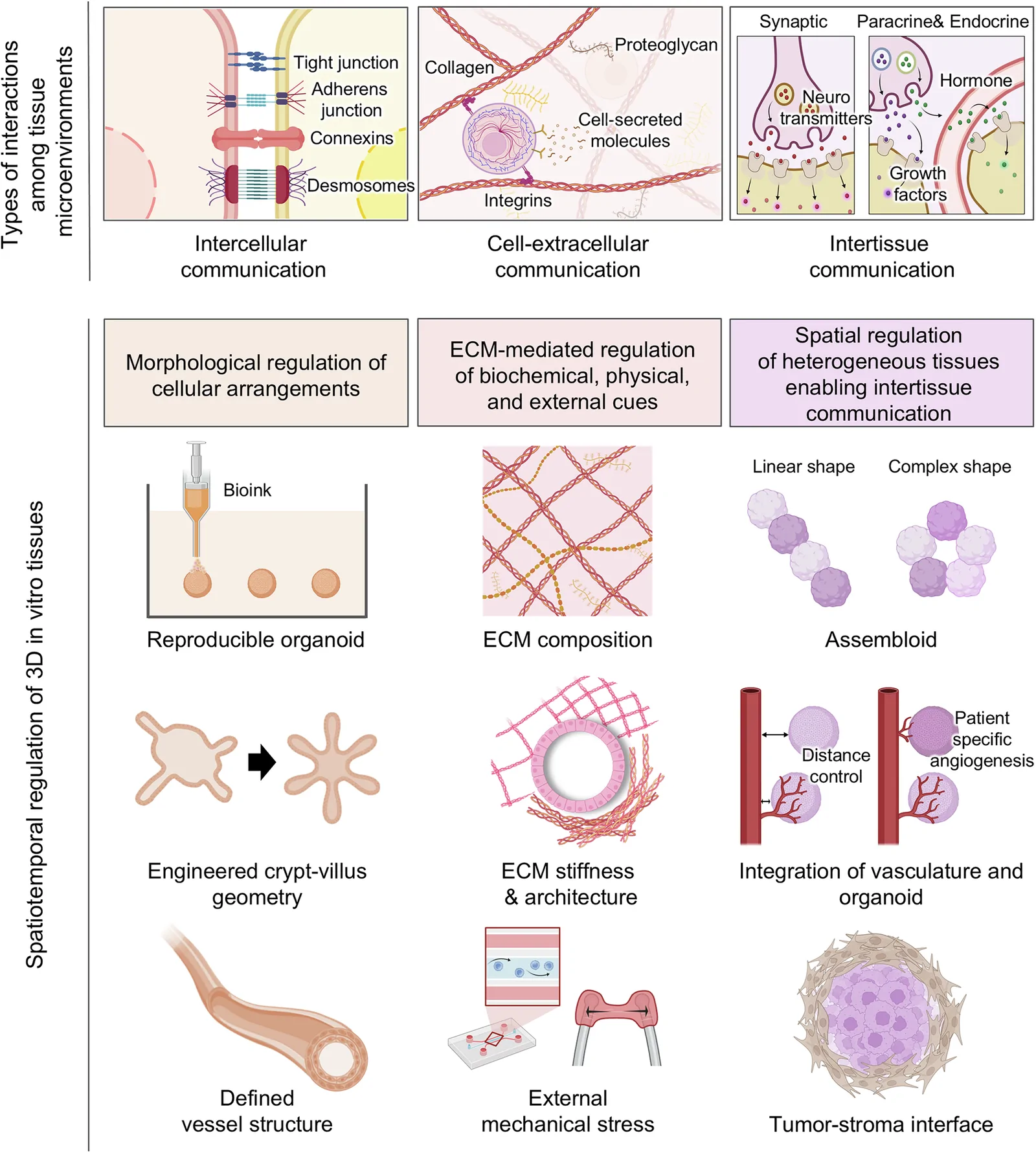
Schematic overview of the spatiotemporal regulation of 3D in vitro tissues to dissect biological crosstalk among tissue microenvironments.

To systematically analyze how biofabrication-based spatiotemporal organization shapes the interactions between cells and extracellular structures, it is necessary to first define the biofabrication techniques employed to control cellular organization and major interaction modes through which cells exchange information. This review surveys key biofabrication techniques and their application for the spatiotemporal modulation of tissue organization. Then, multiscale biological crosstalk is categorized into three principal modes: intercellular, cell–extracellular, and intertissue communication. Intercellular communication encompasses direct and short-range interactions between neighboring cells, including cell–cell junctions, contact-dependent signaling, and locally confined paracrine exchange. Cell–extracellular communication describes how cells sense, remodel, and respond to their surrounding microenvironment, from ECM composition, architecture, and mechanics to externally imposed physical cues such as fluid flow, shear stress, and electric fields. Intertissue communication extends these principles to higher-order tissue interfaces, in which biofabrication allows the spatial integration of distinct tissue compartments and the controlled study of vascular, immune, and stromal cell infiltration as well as soluble, mechanical, and cellular signaling across tissue boundaries. We further discuss how biofabrication-enabled spatiotemporal control can be used to reconstruct and perturb each mode, thereby advancing mechanistic understanding of biological crosstalk in both physiological and pathological contexts. Here, we focus particularly on cancer, where malignant progression unfolds through direct contact among cancer, stromal, and immune cells, signaling through the surrounding ECM, and paracrine crosstalk across the tumor and its surrounding tissue (Fig. 1). By integrating concepts from cell biology, biomaterials science, and biofabrication engineering, this review highlights emerging strategies for building spatially defined in vitro tissue models that enable systematic interrogation of biological crosstalk. Ultimately, these approaches may facilitate the development of more reproducible and predictive experimental systems for functional tissue engineering, disease modeling, and therapeutic discovery^9^.

## Controlling cellular organization in various biofabrication techniques

Key determinants of tissue behavior (e.g., intercellular distance, tissue geometry, mechanical anisotropy, and multicellular boundaries) are best recapitulated through precise control of spatial organization. As discussed above, limitations in organoid self-organization motivate biofabrication strategies that achieve deterministic placement of cells, matrices, and tissue modules across multiple length scales, thereby reconstructing defined biological crosstalk for systematic investigation. Biofabrication is an automated process for fabricating a biologically functional construct, engineered tissue, with a defined structural organization. Various living and non-living materials can be combined—including cells, bioactive molecules, biomaterials, cell aggregates, and cell–material hybrid constructs—through a number of biofabrication techniques^10^. Recapitulating complex multiscale biological crosstalk requires biofabrication strategies that can control tissue organization across multiple hierarchical levels, from the micrometer-scale environment to the spatial positioning of multiple cell types and the assembly of tissue-level architecture. We therefore focus on three representative biofabrication modalities—micromolding, bioprinting, and bioassembly—as complementary approaches for engineering and interrogating biological crosstalk across these scales. For broader coverage of additional biofabrication techniques, previous reviews provide more comprehensive surveys of the field^11–13^. Because each biofabrication technique operates through distinct physical principles with different levels of spatial control, engineered tissue performance depends on how the tissue is fabricated rather than the target design alone. A detailed understanding of each biofabrication technique is therefore essential for the rational selection of fabrication strategies and ultimately the adequate reproduction of intended biological crosstalk.

### Micromolding

Micromolding is one of the foundational biofabrication strategies for engineering spatially defined living constructs, particularly when high structural fidelity must be achieved under cell-compatible conditions. In micromolding, a predesigned template is first fabricated, and then biomaterials, with or without cells, are inserted, thereby enabling the formation of complex architectures with minimal mechanical insult to fragile cells and matrices. One representative approach is based on elastomeric molds fabricated from polydimethylsiloxane (PDMS), which offer high resolution, optical transparency, and ease of pattern transfer. Using soft lithography, PDMS molds can be engineered with microscale grooves, ridges, and chambers that precisely dictate cell organization and tissue geometry^14^. For example, microgrooved PDMS substrates are widely used to induce myocyte or neuronal cell alignment by providing contact guidance cues^15^, while chamber-based designs enable the controlled placement of cell-laden bioinks within predefined volumes and dimensions^16,17^. These features allow researchers to decouple geometric constraints from material deposition, ensuring the reproducible formation of anisotropic or compartmentalized tissues. PDMS-based micromolding is also widely used to develop microphysiological systems (MPSs), including organ-on-a-chip platforms^14^, in which micromolded PDMS devices incorporate interconnected microchannels for the precise control of fluid flow, shear stress, and biochemical gradients across engineered tissues.

While PDMS structures can define the overall architecture and fluidic environment, micromolding is also widely used for hydrogel systems that serve as the primary cellular matrix. Hydrogels, such as collagen and gelatin methacryloyl (GelMA), can be cast within PDMS molds or injected into microfabricated cavities^18^, where subsequent crosslinking stabilizes the construct and preserves predefined structures. In addition, a sacrificial material such as PVA or PF-127 can be integrated into the mold, thereby enabling the formation of perfusable, cell-laden microtissues with controlled geometry and microenvironmental cues^19^. Despite these advantages, micromolding faces several limitations. The demolding process can impose mechanical stress on fragile hydrogel constructs, leading to deformation or structural damage, particularly for high-aspect-ratio or microscale features. Material constraints also arise from the need to balance mold rigidity with release efficiency, often limiting the range of compatible biomaterials and geometries. Furthermore, while micromolding enables the precise fabrication of microstructures, scaling it for large tissue constructs and combining multiple cell types or material layers continues to be challenging^20^.

### Bioprinting

Bioprinting is an additive manufacturing approach that integrates and positions living cells and non-living biomaterials using computer-aided systems to construct heterogeneous, sophisticated engineered tissues^10^. Unlike conventional micromolding approaches that rely on stochastic self-organization, bioprinting provides deterministic control over the positioning of multiple cell types and materials, thereby allowing the reconstruction of complex and heterogeneous tissue architectures^21^. Bioprinting can be classified into inkjet, light-based, and extrusion-based techniques based on the method used to generate each layer. Inkjet bioprinting deposits low-viscosity bioinks in a droplet-by-droplet manner, enabling high-throughput and precise patterning; however, its applicability is limited by narrow viscosity ranges and relatively low cell densities^22^. Light-based bioprinting approaches—including photolithography, digital light processing (DLP), and computational axial lithography (CAL)—utilize photocrosslinkable biomaterials to achieve rapid fabrication and high spatial resolution, often at the expense of material diversity and potential phototoxicity^23,24^. In contrast, extrusion-based bioprinting extrudes continuous filaments of cell-laden bioinks through pneumatic or mechanical forces, making it particularly suitable for high-viscosity materials and high cell densities. While extrusion-based systems offer versatility and scalability, their spatial resolution is typically limited ( > 100 μm), which makes recreating fine microarchitectures, such as capillary networks, challenging^11^.

Across these approaches, the bioprinting process fundamentally relies on three key components: bioinks, printing systems, and crosslinking mechanisms^21^. Bioinks consist of living cells embedded within biomaterial matrices that provide structural support and biochemical cues, thus determining printability and biological functions^25^. Bioinks are typically formulated using synthetic or natural biomaterials, each offering distinct advantages^26^. Synthetic polymers (e.g., polyethylene glycol (PEG)-based hydrogels) provide tunable mechanical properties, controlled crosslinking kinetics, and improved reproducibility, although they often lack intrinsic bioactivity. In contrast, natural matrices (e.g., collagen, gelatin, hyaluronic acid (HA), and decellularized ECM (dECM)) inherently recapitulate tissue-specific extracellular environments and support cell–ECM interactions through integrin-mediated signaling and native biochemical motifs. However, natural hydrogels often exhibit limited mechanical stability, slow or poorly controlled gelation kinetics, and insufficient print fidelity, which restrict their direct applicability in bioprinting. To overcome these limitations, chemical modification strategies have been widely adopted. One example is the methacrylation of gelatin (GelMA), which introduces photocrosslinkable functional groups, enabling rapid and controllable gelation in the presence of photoinitiators (e.g., LAP or Irgacure)^27^. Beyond material composition, bioinks can be further modified by incorporating and blending micro- and nanoscale additives to introduce programmable features, such as tunable crosslinking kinetics, anisotropic architecture, and dynamic remodeling capacity. For instance, incorporating structural and physicochemical modulators (i.e., aligned microfibrous components^28^ or macromolecular crowding agents^29^) induces shear-mediated anisotropy during extrusion and accelerates collagen fibrillogenesis, promoting instant matrix assembly.

Beyond material design, the printing system itself provides an additional layer of control over tissue architecture. Conventional extrusion-based bioprinting faces inherent limitations in fabricating fine microarchitectures ( < 100 μm)^30^. To overcome this challenge, nozzle geometry and flow configuration are modified to form spatially defined, compartmentalized structures. In particular, strategies such as using shape-tunable nozzles and microfluidic-assisted printing allow predefining the spatial distribution of materials prior to deposition, thereby overcoming the intrinsic resolution limits of extrusion systems^31–33^.

### Bioassembly

Bioassembly is the integration of cellular building blocks (e.g., spheroids, organoids, and various-shaped cellular constructs) to reconstruct organomimetic cellular tissue. In contrast to bioprinting, which relies on deterministic external patterning, bioassembly leverages intrinsic cellular processes to drive tissue formation through self-organization^13^. Organoids serve as representative multicellular building blocks that recapitulate key aspects of tissue organization through self-driven morphogenesis, but their limited cellular diversity and diffusion-constrained size restrict their ability to reproduce higher-order tissue architectures, multicellular interactions, and long-range signaling.

To address these limitations, multiple organoids or distinct cell populations are spatially organized and fused to form multi-organoid structures, referred to as assembloids^34^. By physically combining region-specific organoids or introducing stromal and immune components, assembloids allow the study of more complex biological processes^35–42^. For example, integrating motor neuron and skeletal muscle organoids enables the formation of functional neuromuscular junctions for studying synaptic connectivity and activity-dependent signaling^37^, while tumor assembloids incorporating cancer, stromal, and immune components recapitulate tumor–microenvironment interactions, providing insights into immune infiltration, stromal remodeling, and therapeutic response^42^. Each organoid’s organization affects the function of the overall assembloids, as the signal transduction should be in sequence. For instance, assembloids of the ascending neural sensory pathway recapitulate the directional neuronal signal relay from sensory neurons to the cerebral cortex^38^. However, organoid fusion within assembloids is performed by manually positioning organoids close together using low-attachment platforms such as U-bottom wells or shared Matrigel droplets, where fusion occurs through intrinsic cell–cell adhesion. This approach provides only coarse, poorly reproducible control over spatial parameters, including inter-organoid distance, sequence, orientation, interface geometry, and higher-order 3D arrangement. To spatially control the organoids, bioassembly techniques are introduced so that the organoids can be picked and placed through pneumatic pressure^43–45^ or magnetic force^46^.

Despite these advances, assembloids still face scalability and structural robustness limitations, which restrict their ability to reconstruct macroscopic tissue architectures and achieve tissue-level functionality. To overcome these challenges, bioassembly strategies have been extended to integrate cell-laden hydrogels with defined geometries, enabling larger and more functionally relevant tissue constructs to be fabricated^47^. Using micromolding and bioprinting techniques, cell-laden structures with controlled geometries—such as point-, line-, and plane-shaped configurations—can be generated and assembled in a modular manner^48^. For example, to recapitulate tissue-level functions such as the heart’s left ventricular twist, engineered heart tissues (EHTs) with strip and ring geometries have been sequentially assembled into multilayered constructs, resulting in a 3D chamber capable of multiaxial contraction^49^.

## Types of biological crosstalk in tissue microenvironments

Cells never exist as independent organisms; they are constantly in crosstalk with neighboring cells and the surrounding microenvironment. Rather than operating through a single signaling mechanism, cells interpret a diverse set of extracellular inputs that collectively shape intracellular signaling networks and ultimately determine cellular behavior. These inputs arise from direct physical contact between neighboring cells, diffusible biochemical signals that propagate through extracellular space, and interactions with the ECM, which provides both biochemical ligands and mechanical cues. This intercellular and extracellular communication serves as a critical upstream regulator that defines the amplitude, duration, and functional outcome of intracellular signaling events (Box 1). Cells continuously integrate spatially distributed signals to regulate processes such as proliferation, differentiation, migration, and collective tissue organization. Therefore, these interactions should be studied to elucidate how cellular signaling is organized across tissues. In this section, we classify biological crosstalk within tissue microenvironments into three principal types of communication: intercellular, cell–extracellular, and intertissue communication. We then discuss how these mechanisms operate under physiological conditions and how their dysregulation contributes to pathological states such as cancer. These three principal interactions will help clarify the effects of biofabrication parameters, thereby providing a guideline for biofabricated tissue design.

Box 1: core intracellular signaling pathways mediating biological crosstalk Spatiotemporal signaling regulates cell fate, organization, and function and is orchestrated through a network of highly conserved pathways, including the Wnt/β-catenin, Notch, TGF-β/BMP, FGF, and Hippo/YAP–TAZ pathways. The Wnt pathway, comprising canonical (β-catenin–dependent) and non-canonical branches, governs fundamental processes such as cell proliferation, polarity, and migration and is essential for maintaining stem cell renewal across multiple tissues^189^. The Notch signaling pathway mediates direct cell–cell communication, thereby controlling the self-renewal, differentiation, and dedifferentiation of stem and progenitor cells. Although Notch activity is high during embryogenesis, it remains low in mature tissues and rapidly reactivates upon injury or stress, contributing critically to tissue repair and regeneration^190^. The TGF-β signaling network plays a context-dependent role encompassing cell survival, growth, metabolism, differentiation, adhesion, and apoptosis. Within this superfamily, BMPs serve as key morphogens in embryonic patterning and lineage specification^191,192^. Another central regulator of pluripotency and development, the FGF pathway, influences cell proliferation, migration, differentiation, organogenesis, and tissue repair through precise spatial and temporal activation^193^. Finally, the Hippo pathway, mediated by the transcriptional co-activators YAP and TAZ, integrates multiple upstream cues—cell density, polarity, mechanical stress, and soluble factors—to modulate cell proliferation, apoptosis, and stem cell self-renewal^194^. Collectively, these pathways form an interconnected signaling network that orchestrates dynamic cellular interactions and structural organization within 3D tissue models and organoids.

Building on these developmental and homeostatic functions, many conserved signaling mechanisms are pathologically rewired in clinical conditions such as cancer. Canonical intracellular pathways such as TGF-β/SMAD, JAK–STAT, PI3K–AKT, MAPK/ERK, NF-κB, Notch, and Wnt/β-catenin represent the core regulatory architectures through which tumor cells interpret and execute proliferative, invasive, and survival programs. Among these, the TGF-β/SMAD axis plays a dual role: while acting as a tumor suppressor in early carcinogenesis, it becomes a potent driver of EMT, invasion, CAF activation, and immune evasion in advanced tumors^195^. The JAK–STAT pathway, particularly STAT3, is frequently activated by cytokines originating from CAFs and TAMs, promoting stemness, chronic inflammation, and T cell suppression^196^. Similarly, the PI3K–AKT and MAPK/ERK cascades are among the most pervasive oncogenic circuits, mediating growth factor–driven proliferation, survival, motility, and metabolic adaptation. Their sustained activation often results from oncogenic mutations (e.g., KRAS, PIK3CA) or tumor suppressor loss^197,198^. The NF-κB pathway integrates inflammatory signals from both the tumor and stromal compartments, reinforcing a feedforward loop of cytokine production, immune suppression, and enhanced metastatic potential^199^. In addition, developmental pathways such as Notch and Wnt/β-catenin confer stem-like traits, lineage plasticity, and resistance to therapy. While Notch signaling regulates angiogenesis and cell fate decisions, Wnt ligands derived from CAFs or the ECM microenvironment support cancer stemness and contribute to the establishment of immune-desert phenotypes^200,201^. Together with these canonical routes, microenvironment-responsive pathways including hypoxia-induced HIF-1α activation and integrin-FAK-YAP mechanotransduction further expand the signaling landscape through which cancer cells integrate environmental cues to drive malignant phenotypes. The HIF-1α signaling pathway mediates cellular adaptation to microenvironmental hypoxia by stabilizing HIF-1α and inducing transcriptional programs that promote metabolic reprogramming, angiogenesis, and survival^202^. The integrin-FAK-YAP signaling axis transduces mechanical cues from the ECM, such as matrix stiffness, fiber alignment, and tensile forces, into intracellular signals that promote YAP/TAZ nuclear translocation and regulate cytoskeletal remodeling, invasive behavior, and CAF-mediated matrix reinforcement^65^.

### Intercellular communication

Intercellular communication occurs when neighboring cells establish physical contact and transmit localized signals across membranes. These contact-dependent interactions are essential for coordinating multicellular organization, and they are mediated through specialized junctional complexes (e.g., adherens, tight, and gap junctions) as well as juxtacrine signaling systems.

Cadherin-based adherens junctions—mediated by E-cadherin in epithelial tissues, N-cadherin in neural/mesenchymal contexts, and VE-cadherin in endothelial cells—form Ca²⁺-dependent homophilic cell–cell adhesions that are mechanically and biochemically coupled to intracellular signaling. Upon engagement, cadherins connect to β- and α-catenin, thereby linking junctional complexes to the actin cytoskeleton. Adherens junctions not only establish cohesive epithelial sheets and apico–basal polarity through this architecture but also enable force transmission across multicellular assemblies, supporting collective tissue mechanics^50^. Importantly, cadherin complexes can regulate β-catenin availability, providing a direct interface between junctional state and Wnt pathway crosstalk^51^.

Tight junctions provide an additional layer of contact-dependent communication by defining selective epithelial barriers and compartment boundaries. These junctions are built from claudins, occludin, and junctional adhesion molecules (JAMs), which are scaffolded by cytoplasmic adaptors such as ZO-1/2/3^52^. Mechanistically, they seal the paracellular space, controlling ion and solute permeability between neighboring cells. Functionally, they are essential for maintaining epithelial polarity by enforcing separation between apical and basolateral domains while simultaneously regulating tissue-level signaling by restricting or permitting paracellular diffusion of morphogens between compartments^53^. In contrast to barrier-forming junctions, gap junctions act as direct conduits for intercellular signal propagation by allowing cytoplasmic continuity at the small messenger level^54^.

Gap junction channels are assembled from connexins and permit the exchange of ions and small metabolites such as cAMP and ATP, thereby coupling neighboring cells electrically and metabolically^55^. This direct exchange supports the synchronization of electrical and metabolic activity across local cell populations. Beyond junctional complexes, direct cell–cell communication is also mediated by juxtacrine signaling, in which membrane-tethered ligands and receptors require direct contact to activate one another. Two major examples are Notch–Delta/Jagged and Eph–ephrin signaling^56^. The former provides a contact-dependent mechanism to determine stem versus differentiated fate, whereas the latter coordinates repulsion and adhesion at tissue boundaries, enabling compartmentalization and pattern formation.

### Cell-extracellular communication

Cells also communicate with their “house,” the ECM, a dense network of molecules that lies between cells, primarily composed of structural proteins (e.g., collagens, elastin), adhesive glycoproteins (e.g., fibronectin, laminin, vitronectin), and proteoglycans/glycosaminoglycans (GAGs) (e.g., HA and heparan sulfate)^57^. Cells attach to ECM components mainly through cell surface receptors such as integrins, interpret biochemical and mechanical signals, and convert them into intracellular responses that determine cell morphology and fate^58,59^. For example, integrins are heterodimeric transmembrane proteins that bind to specific peptide motifs within ECM ligands such as the RGD sequence (Arg-Gly-Asp)^60^. Upon ligand binding, integrins undergo conformational activation and cluster into focal adhesions, where they connect the ECM to the actin cytoskeleton via adaptor proteins like talin and vinculin. Through this molecular linkage, cells exert traction forces on the ECM to probe its mechanical properties, such as stiffness, topography, and viscoelasticity, thereby initiating mechanosensing^61^. Concurrently, autophosphorylation activates focal adhesion kinase (FAK) and Src, initiating downstream signaling cascades such as RhoA/ROCK-mediated cytoskeletal remodeling, MAPK/ERK, PI3K/AKT-mediated survival and proliferation, and YAP/TAZ nuclear translocation^62–64^. For instance, increased ECM rigidity promotes the nuclear localization of YAP/TAZ and induces osteogenic or myofibroblastic differentiation, whereas softer matrices maintain a quiescent or neurogenic phenotype^65,66^.

While the ECM instructs cellular behavior, cells continuously remodel the ECM by secreting matrix proteins and matrix-remodeling enzymes, establishing a bidirectional feedback loop. Fibroblasts and myofibroblasts deposit structural proteins such as collagen I, fibronectin, and laminin, while matrix metalloproteinases (MMPs) and cathepsins degrade ECM components to allow turnover and migration. Additionally, lysyl oxidase (LOX) and transglutaminase 2 (TG2) catalyze the covalent crosslinking of collagen and elastin, increasing matrix stiffness. This remodeling alters ECM composition, crosslinking density, architecture, and mechanical properties, thereby feeding back to influence cellular mechanosensing and signaling^67^.

Beyond the static properties of matrix composition and stiffness, cells within living tissues are constantly exposed to dynamic mechanical forces that profoundly influence their behavior. For example, the endothelial cells that compose the vasculature are classical shear-responsive cells: laminar shear stress maintains their quiescent, anti-inflammatory phenotype, while disturbed or oscillatory flow induces pro-inflammatory signaling (NF-κB activation, VCAM-1 expression). Beyond the vasculature, interstitial flow (IF) within 3D matrices exerts subtle shear forces (0.1–10 dyn/cm²) on stromal and immune cells. This flow not only causes the convection of soluble factors and chemokines but also reorients collagen fibers, establishes pressure gradients, and activates mechanosensitive ion channels (Piezo1, TRPV4) and integrin–RhoA–YAP signaling in fibroblasts, epithelial, and immune cells^68^. Solid stress and fluid shear are mechanically coupled through ECM deformation and poroelasticity, creating a multiphase mechanical system in which matrix strain, pore size, and fluid mobility are interdependent. For instance, compressive solid stress increases local interstitial fluid pressure (IFP) and reduces IF, while enzymatic ECM degradation transiently relieves pressure and restores perfusion.

### Intertissue communication

Intertissue communication involves local communication over short distances (paracrine and synaptic signaling) or long distances via hormones (endocrine signaling). Paracrine signaling enables long-range coordination by secreting and diffusing soluble factors—including growth factors, cytokines, and morphogens—into the extracellular space. Classic developmental morphogens such as fibroblast growth factor (FGF), Wnt, bone morphogenetic protein (BMP), sonic hedgehog (SHH), and vascular endothelial growth factor (VEGF) function paracrinally to regulate proliferation, fate specification, and tissue patterning^69^. Paracrine signals can generate local gradients that encode positional identity (e.g., along anterior–posterior or dorsal–ventral axes), maintain stem cell niches, and synchronize responses across nearby cell populations. These signals’ spatial distribution is further shaped by ligand diffusion, receptor-mediated uptake, ECM binding, and enzymatic degradation, collectively determining the range and persistence of signaling gradients^70^. For example, heparan sulfate proteoglycans within the ECM can sequester morphogens such as FGF and Wnt, modulating their effective diffusion and signaling strength^71^.

In excitable tissues such as the nervous system, synaptic signaling represents a specialized form of highly localized indirect communication. In this process, neurotransmitters—including glutamate, GABA, and dopamine—are released from presynaptic terminals into the synaptic cleft^72^ and bind to ligand-gated ion channels or G-protein–coupled receptors on postsynaptic cells^73^. This rapid and spatially confined signaling enables millisecond-scale communication and precise information transfer across neuronal circuits.

For long-distance communication between different organs, specialized cells release hormones into the bloodstream, which move to a distant target to elicit a response. Endocrine signaling enables systemic coordination of physiological processes such as metabolism, growth, reproduction, and immune regulation^74^. Classic examples include insulin and glucagon regulating glucose homeostasis, steroid hormones modulating transcriptional programs through nuclear receptors, and cytokines such as interleukins coordinating immune responses across tissues^75^. Together, paracrine, synaptic, and endocrine signaling establish multilayered communication networks that allow cells to integrate local microenvironmental cues with organism-level physiological regulation.

### Current approaches to recreating biological crosstalk in a physiological context

A healthy organism provides cells with a continuously regulated microenvironment of biochemical and biophysical cues that collectively sustain tissue homeostasis. In contrast, cells cultured in vitro lack these native inputs, and the resulting loss or distortion of contextual signals can reshape downstream transcriptional and translational programs, leading to altered phenotype, functional decline, or apoptosis. To mitigate these effects, culture media are commonly supplemented with defined soluble factors that support cell survival and preserve cell-state identity. For example, Rho-associated kinase (ROCK) inhibitors improve the survival of dissociated human pluripotent stem cells (hPSCs) by suppressing anoikis^76^, while basic FGF (bFGF/FGF2) stabilizes pluripotency-associated signaling and supports self-renewal during expansion^77^. For primary cell cultures, which often exhibit rapid functional deterioration after isolation, lineage-appropriate “maintenance” cues are similarly incorporated to preserve specialized activity (e.g., VEGF-A to support endothelial survival and phenotype^78^ or insulin to sustain metabolic and contractile programs in cardiomyocytes^79^). In parallel, culture dishes are often coated with ECM proteins (e.g., collagen or laminin) or complex matrices (e.g., Matrigel) to provide integrin-binding sites and partially restore the tissue-like adhesive and mechanical cues that regulate cytoskeletal organization and mechanotransduction.

Recapitulating intercellular interactions is also critical for directing stem cell differentiation. In most differentiation workflows, each developmental transition is implemented by temporally controlled activation or inhibition of identified signaling pathways. Mesoderm induction, for instance, is commonly achieved by activating canonical Wnt and BMP signaling using small molecules and growth factors such as CHIR99021 (a GSK3 inhibitor that stabilizes β-catenin) and BMP4^80^. Definitive endoderm specification typically relies on strong TGF-β/Activin/Nodal pathway activation using Activin A to drive SMAD2/3 signaling. In contrast, ectodermal (particularly neural) induction is frequently promoted by suppressing BMP signaling using inhibitors such as LDN-193189, often combined with TGF-β pathway inhibition^81^. Importantly, however, adding exogenous soluble factors, even in carefully timed sequences, captures only part of the developmental progress. During embryonic development, the spatiotemporal environment plays a major role in the pathway activity, including tissue geometry, cell density, polarity, and localized ligand presentation, the combination of which generates gradients, feedback, and contact-dependent signaling.

Organoids partially restore these spatial constraints by self-organizing into three-dimensional (3D), high-density cellular architectures, facilitating cellular hierarchy formation. Thus, they often develop heterogeneous yet organized populations spanning multiple lineages and maturation states, which support more physiologic cell–cell junctional networks and juxtacrine signaling than is typically achievable in dispersed 2D cultures. However, organoid architectures are usually constrained to near-spherical morphologies arising from isotropic growth, limiting their ability to reproduce the anisotropic geometries characteristic of many native tissues. Their growth is also often restricted by the diffusion-limited transport of oxygen and nutrients, which constrains organoid size and can lead to hypoxia or necrotic cores in larger constructs.

### Current approaches to recreating biological crosstalk in a pathological context

Pathological conditions such as cancer, fibrosis, and chronic inflammatory disorders arise from dysregulated interactions among multiple cell types rather than defects confined to individual cells. Disease progression, therefore, involves the alteration of cellular states together with the disruption of coordinated biological crosstalk across heterogeneous populations. Among these conditions, tumors best exemplify this principle, as malignant progression depends on structured interactions among cancer cells, stromal cells, infiltrated immune cells, and the ECM^82,83^. Hence, this review focuses on tumors as a representative pathological system to examine intercellular interactions in a disease context.

Epithelial–mesenchymal transition (EMT), a key event in tumor progression, is initiated by the disruption of direct cell–cell adhesion^84^. Transcriptional repressors such as Snail and Twist suppress E-cadherin expression, weakening adherens junctions and compromising epithelial integrity. Concurrent disassembly of tight junctions alters apicobasal polarity and redistributes membrane-associated signaling complexes, changing how tumor cells respond to external stimuli and facilitating invasive behavior^85^.

Loss of epithelial cohesion is accompanied by dynamic ECM remodeling. Through integrin-based adhesion complexes, cells sense matrix stiffness and composition and activate mechanotransduction pathways, including FAK, Src, and YAP/TAZ signaling. Tumor cells activate resident fibroblasts through paracrine signaling^86^—particularly via factors such as TGF-β—driving their transition into cancer-associated fibroblasts (CAFs)^87^. Subsequently, these CAFs deposit matrix proteins and secrete MMPs, remodeling the ECM and altering tissue stiffness, IFP, and matrix architecture^88^. These changes promote cancer invasion, influence vascular organization, and limit drug penetration and immune infiltration^89^.

At larger spatial scales, diffusible signals coordinate interactions among tumor, stromal, and endothelial cells. Growth factors and cytokines—including VEGF, interleukins, and CCL/CXCL family members—regulate angiogenesis and immune cell recruitment. Persistent VEGF signaling induces the formation of structurally and functionally abnormal tumor vessels characterized by leakiness, poor perfusion, and heterogeneous oxygen distribution^90^. The resulting hypoxia stabilizes HIF-1α, enhancing glycolytic metabolism and metastatic potential^91^. Signaling gradients also shape immune composition within the tumor microenvironment (TME)^92^. Chemokines recruit myeloid and lymphoid populations, yet sustained exposure to TGF-β, IL-10, and checkpoint ligands promotes immunosuppressive phenotypes and attenuates cytotoxic T cell activity. In parallel, abnormal vasculature and dense ECM structures restrict immune cell infiltration and spatial distribution.

As primary tumors evolve under mechanical and metabolic stress^93^, a subset of cells acquires dissemination capacity. Circulating tumor cells (CTCs) adhere to endothelial adhesion molecules such as ICAM and VCAM via integrins, enabling vascular arrest and transendothelial migration. Distant organs’ successful colonization depends on interactions with local stromal cells and ECM niches that provide survival and proliferative signals. Collectively, tumor progression reflects coordinated alterations in direct cell–cell contact, matrix engagement, and long-range signaling rather than a simple linear sequence of events^94^.

## Biofabrication-enabled reconstruction of multiscale biological crosstalk

We have explored how native tissue cells communicate with one another through intercellular, cell-extracellular, and intertissue communication. As discussed in Sections “ Introduction” and “Controlling Cellular Organization in Various Biofabrication Techniques”, the intrinsic limitations of organoid self-organization have motivated the adoption of biofabrication technologies to provide control over spatiotemporal cues that could not be achieved through organoid culture alone. Here, we categorize spatiotemporal regulatory strategies into three levels corresponding to the principal modes of biological crosstalk: morphological regulation of cellular arrangements, regulation of the microenvironment via the ECM, and regulation of multicellular tissue integration. Together, these approaches illustrate how biofabrication technologies can systematically manipulate spatiotemporal tissue organization to facilitate the investigation of biological crosstalk in both physiological and pathological contexts, with particular emphasis on tumor-specific dynamics within the TME.

### Biofabrication-enabled morphological organization of cellular arrangements

The morphological organization of cells within tissues fundamentally determines their interactions, particularly through direct cell–cell contact. Biofabrication technologies enable the deliberate arrangement of cells into specific geometries, from spheroidal constructs like organoids to more complex architectures like the intestine’s crypt-villus structure, vasculature tubular structures, and cancer tissue with compartmentalized cell distribution. These morphologically defined constructs elucidate how cell density, shape, and spatial juxtaposition regulate contact-dependent signaling, offering new insights into developmental processes and tumor biology.

To engineer tissues with structural complexity approaching native organization, many biofabrication workflows begin by dispersing cells into single-cell suspensions and depositing them within a lumen or throughout a 3D matrix. During subsequent culture, cells generate autocrine and paracrine cues and actively reorganize through migration and adhesion-driven self-assembly. As intercellular spacing decreases, canonical cytoskeletal pathways, including PI3K–Rac1-mediated protrusion and RhoA–ROCK-dependent contractility, promote chemotactic and contact-seeking behaviors^95^. Once cells establish physical contact, cadherin-based adherens junctions stabilize cell–cell adhesion and couple junctions to the actin cytoskeleton, enabling contact-dependent signaling and force transmission that support tissue-level coordination^96^. Because the probability, timing, and topology of junction formation are heavily dependent on initial cell–cell distance and neighborhood composition, early spatial organization control is vital to reproducible tissue assembly and maturation.

**Fig. 2:**
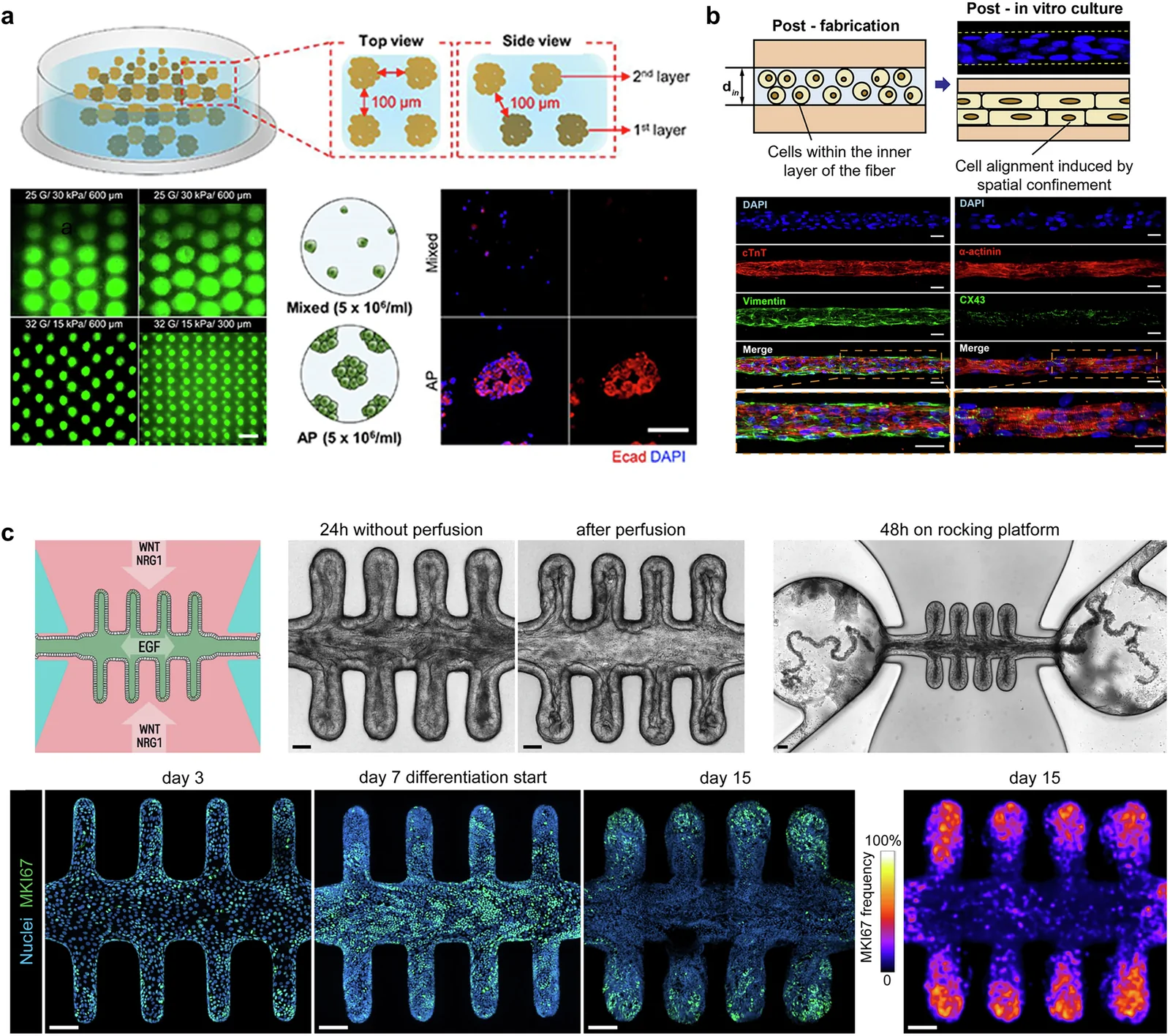
Physiological examples in morphological regulation of cellular arrangements. **a** Embedded bioprinting of pancreatic beta cells and their accumulation with high cell density. Reproduced with permission^97^, Copyright 2021, IOP Publishing Ltd. **b** Formation of aligned cardiac tissue through migration and contraction, following formation of gap junctions (Cx43). Reproduced with permission^102^, Copyright 2025, Elsevier Ltd. **c** Colon tissue with crypt-villus geometry, with high proliferation (MKI67 + ) in the crypt region. Reproduced with permission^188^, Copyright 2024, Elsevier Ltd.

Embedded 3D bioprinting with cell-laden bioinks provides a practical route to impose this control during fabrication. In this approach, a supportive bath matrix mechanically stabilizes the extruded filaments, allowing cells to be positioned along predefined paths and at prescribed densities, thereby setting initial intercellular distances and local multicellular geometry. By programming deposition patterns, embedded printing can generate diverse tissue architectures—including spheroidal aggregates resembling pancreatic islets^97,98^ and tubular vascular structures from endothelial cells^99,100^—while maintaining a permissive 3D microenvironment for subsequent self-organization. Moreover, initial cadherin-mediated assembly promotes junctional communication over time, as gap junction connectivity typically increases during culture, enabling direct intercellular exchange of ions and small molecules and providing an additional layer of tissue synchronization. For example, Hwang et al. bioprinted pancreatic β cells within a dECM-based material, where printing parameters enabled control over the size and number of spheroidal structures (Fig. 2a). Importantly, printing-established proximity promoted rapid formation of E-cadherin-positive cell–cell junctions, consistent with accelerated aggregate assembly. Compared with a non-patterned “mixed” condition with a matched total cell number, the printed configuration preferentially supported islet-like organization and increased the expression of maturation-associated genes. These cell–cell adhesions further promoted the development of gap junctions (connexin 36), ensuring high functionality after the maturation of engineered tissue. Similarly, EHT can be fabricated by molding cardiomyocyte-laden bioink between two anchors, controlling the dimensions of the mold and anchors through 3D printing^49^. After the cells’ initial migration and contraction, gap junctions, including connexin 43, facilitate the functional maturation of electrical signal propagation (Fig. 2b). To create a larger construct that mimics the left ventricle’s chamber shape, the EHTs are used as building blocks and fused together. The chamber-like structure can also be fabricated using embedded 3D printing^101,102^, where the cardiomyocytes are aligned and form gap junctions, showing electrical signal propagation that circulates around the chamber structure resembling that of native tissue.

The same design logic applies to vascular morphogenesis. When endothelial cells are printed as linear features within a supportive matrix, collective migration and remodeling can generate a tubular lumen, accompanied by the maturation of endothelial junctional markers, such as VE-cadherin. Moreover, printing-defined geometry offers a controllable handle to introduce structural features—including stenoses or tortuosity—by modifying path design and writing speed, thereby tuning flow profiles and shear stresses once perfusion is introduced^31,103^. Thus, biofabrication can be used to not only initiate tissue formation by controlling early contact topology but also create conditions that support the progressive maturation of multicellular communication networks.

Finally, tissue architectures regulate signaling through not only cell–cell contact probability but also mechanotransduction and spatially patterned morphogen exposure. The small intestinal epithelium exemplifies this principle: the crypt–villus geometry supports compartmentalized signaling environments in which stem and progenitor cells reside in crypt regions and differentiate as they migrate toward villi, coordinated by pathways such as Wnt and BMP signaling (Fig. 2c). Consistent with a geometry-driven mechanism, Nikolaev et al. developed a hydrogel-based microdevice containing an open microchannel with a crypt–villus-like topography. Seeding intestinal stem cells into this architecture yielded region-specific differences in lineage composition, with enrichment of SOX9-positive cells in crypt-like domains, illustrating how engineered geometry and matrix boundary conditions can be leveraged to interrogate the coupled biochemical and mechanical regulation of cell fate^104^.

Taken together, biofabrication provides a powerful means to regulate intercellular communication by defining the initial spatial organization of cells and guiding their subsequent self-assembly into functional tissue architectures. In the short term, controlling cell density, spacing, and anisotropic distribution within a 3D matrix determines the probability and topology of early cell–cell contact. During culture, these initially patterned constructs undergo further reorganization, which can be directed by biofabricated scaffolds and boundary conditions. Engineered tissues progressively establish adherens junctions, gap junctions, and other communication networks that support cellular maturation and tissue-level coordination through this process. However, many current fabrication strategies require dissociation of cells into single-cell suspensions, temporarily disrupting pre-existing intercellular communication and delaying the emergence of coordinated multicellular behavior. To overcome this limitation, recent approaches have begun to incorporate pre-assembled multicellular units, such as spheroids^105^ and microtissues^106^, as biofabrication building blocks. These strategies may preserve early-stage cell–cell communication while enabling higher-order spatial organization, offering a promising route toward constructing more mature and physiologically relevant in vitro tissues.

The morphological organization of cellular arrangements also plays a critical role in cancer models. Conventional 2D culture inherently lacks tissue-level architecture, and even tumor organoids rely on stochastic self-assembly, such that the resulting tissue geometry is governed largely by probability rather than design. Bioprinting overcomes this constraint by enabling cells to be deposited directly into a prescribed geometry, allowing the fabrication of uniform organoids and complex architectures that self-assembly alone cannot reliably achieve. In doing so, bioprinting converts tissue geometry from an uncontrolled outcome of self-organization into a defined experimental variable. Because organoid shape no longer varies stochastically across replicates, AI-based image analysis can attribute subsequent changes in growth or morphology to the experimental condition itself rather than to baseline geometric variation. Likewise, fixing vascular curvature independently of the cellular remodeling that ordinarily generates it isolates curvature as an experimental variable.

**Fig. 3:**
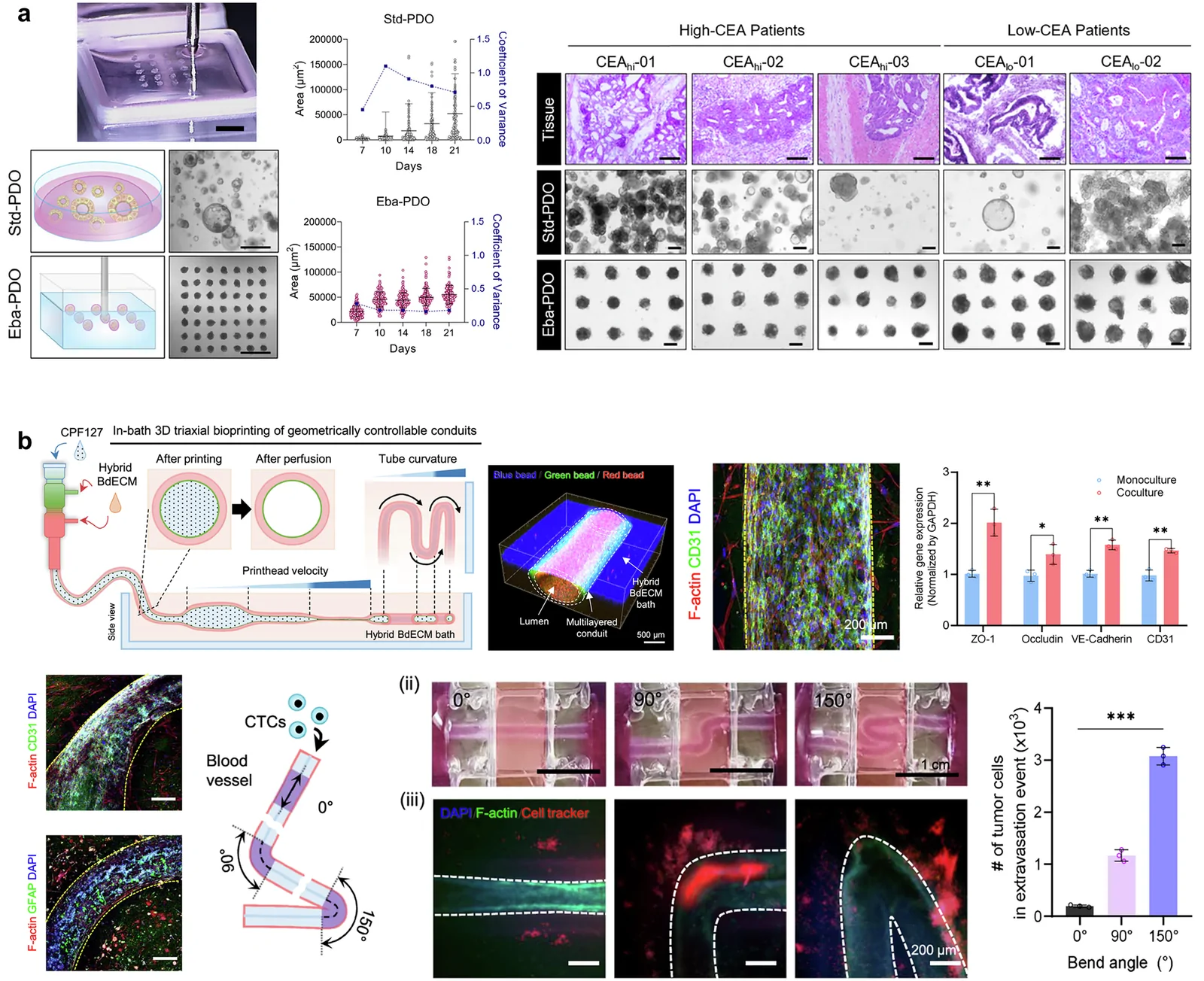
Pathological examples in morphological regulation of cellular arrangements. **a** Embedded bioprinting of uniform arrays of cancer organoids. Reproduced with permission^110^, Copyright 2022, Wiley–VCH GmbH. **b** Tri-axial bioprinting of multi-layered and occluded vasculature interacting with circulating tumor cells. Reproduced with permission^31^ Copyright 2023, Springer Nature.

In the tumor patient-derived organoid (PDO) model, tumor cells are dissociated from a patient’s tumor tissue and embedded in 3D ECM hydrogels, where the dissociated cells self-assemble stochastically over 7–14 days into organoids with highly heterogeneous sizes and shapes^107,108^. The structural organization of tumor PDOs is a critical determinant of experimental consistency, because uncontrolled organoid geometry can alter epithelial packing and junctional architecture, thereby confounding tumor behavior analysis^109^. To address this, Han et al. exploited an embedded bioprinting technique to fabricate uniform arrays of colorectal tumor organoids by depositing high-density tumor cells at defined locations and in predefined volumes within a supporting bath (Fig. 3a)^110^. Compared with conventional PDO culture, the bioprinted organoids exhibited enhanced reproducibility and structural uniformity, thereby facilitating the controlled formation of cadherin-based adherens junctions and tight junction complexes across tumor organoids. Combined with AI-based image analysis, this structural uniformity enabled the automated and quantitative assessment of growth kinetics and morphological changes, establishing organoid geometry as a defined experimental variable rather than an uncontrolled source of variability in tumor modeling.

Direct tumor cell–vascular endothelial cell interactions are key regulators of tumor progression, as they influence endothelial barrier remodeling and extravasation of cancer cells within the TME^111^. To reconstruct such interactions in vitro, researchers have developed perfusable tubular vascular models using post-seeding approaches in microfluidic devices or multiaxial nozzle bioprinting, thereby enabling tumor cells to interface directly with 3D vessel walls^112,113^. Building on this biofabrication framework, Park et al. combined triaxial bioprinting with embedded bioprinting to fabricate multilayered cerebrovascular conduits composed of endothelial cells and pericytes. This study showed that including pericytes enhanced vascular cell–cell interactions compared with vasculatures formed solely by endothelial cells, as evidenced by the upregulation of junctional markers of ZO-1, Occludin, and VE-Cadherin (Fig. 3b)^31^. Furthermore, by exploiting the design freedom of bioprinting to vary printing speed and path, they reproduced occluded or morphologically altered vascular structures, which allowed them to investigate how vessel curvature regulates CTC extravasation. This study revealed that larger vessel curvature prolonged the dwell time of cancer cells near the endothelium, thereby increasing contact-dependent adhesion events. This response was associated with the elevated expression of E-selectin, VCAM-1, and ICAM-1, together with decreased VE-Cadherin expression, indicating endothelial junction remodeling during cancer cell–endothelium interactions.

However, the structural uniformity achieved through biofabrication may not necessarily mean a biologically faithful one. For example, intertumoral heterogeneity across patients and intratumoral heterogeneity within a single tumor are both established correlates of drug response and invasiveness in clinical tumors^83^, so a deliberately uniform construct may discard exactly the variation a given study is meant to capture. Furthermore, the specific geometries imposed by biofabrication let researchers isolate geometry as the variable driving the observed tumor behavior, but do not establish that the geometry tested is representative of the range actually found across patient tumors and vasculature.

### ECM-mediated regulation of biochemical, biophysical, and external cues

ECM composition governs integrin engagement, ligand density, protease sensitivity, and growth factor sequestration, all of which shape downstream signaling and lineage specification. Cells continuously deposit, degrade, and remodel their surrounding ECM, but this endogenous remodeling requires time, and cells are highly sensitive to their initial microenvironment’s biochemical and mechanical properties. If the surrounding matrix fails to provide appropriate adhesive ligands, stiffness, or structural support during early culture, cells may undergo apoptosis, lose function, or differentiate to unintended lineages. Hence, cells are commonly embedded in ECM-derived materials such as collagen, Matrigel, or dECM^114^. Although these materials can provide a permissive environment for cell survival, biofabrication expands the role of the matrix from a passive structural support to an active, instructive regulator of cell fate and tissue organization.

**Fig. 4:**
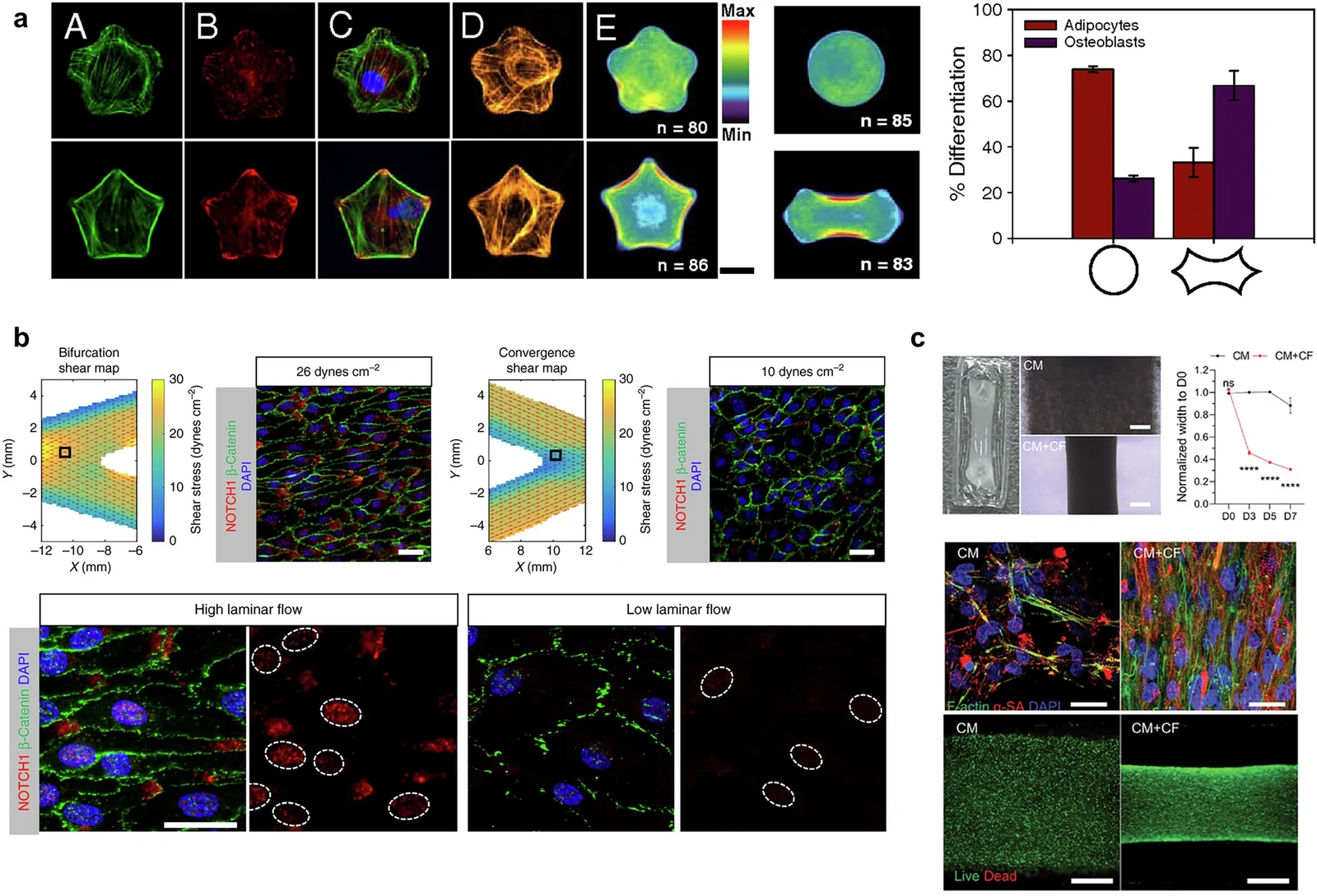
Physiological examples in ECM-mediated regulation of biochemical, biophysical, and external cues. **a** (Left) Structure of cells in the flower and star shapes. F-actin (green), vinculin (red), nuclei (blue), myosin Iia (yellow). Fluorescent heatmaps of > 80 cells stained for myosin IIa as a quantitative measure of contractility. (Scale bar, 20 μm) (Right) Cell structure controls MSC fate; cells with a holly shape favored osteogenic fate. Reproduced with permission^116^, Copyright 2010, National Academy of Sciences. **b** External flow affects endothelial cell alignment through NOTCH1 and β–catenin, demonstrated through different flow speeds. Reproduced with permission^121^, Copyright 2017, Springer Nature. **c** Cardiac tissue with fibroblast contracts and provides external stress, promoting alignment of the cardiomyocytes. Reproduced with permission^49^, Copyright 2024, Wiley–VCH GmbH.

One major mechanism through which engineered matrices regulate cell behavior is mechanotransduction. Mesenchymal stem cells (MSCs), for example, are known to undergo adipogenic or osteogenic differentiation depending in part on the elastic modulus of the surrounding substrate or matrix^115^. Beyond bulk stiffness, geometric confinement and local curvature also influence lineage commitment by modulating cell spreading, cytoskeletal tension, and nuclear mechanosignaling. Kilian et al. demonstrated that MSCs cultured in adhesive wells with identical areas but different aspect ratios and curvatures exhibited distinct differentiation outcomes, with elongated, highly curved geometries favoring osteogenesis (Fig. 4a). Mechanistically, these effects were associated with enhanced actomyosin tension and activation of RhoA/ROCK-dependent signaling^116^, which, in turn, regulates the nuclear localization of YAP/TAZ^65^. Thus, engineered geometry can convert physical constraints into lineage-specific biochemical responses. While these microscale geometries can direct lineage commitment at the single-cell level, mechanotransduction in the engineered tissue modifies cell behavior through mechanical coupling to a surrounding 3D ECM. The 3D microenvironment distributes adhesive ligands in all directions and transmits forces through a fibrillar architecture that differs from the planar surface. As a result, cell behavior can be controlled by matrix porosity, fiber alignment, viscoelastic relaxation, and matrix geometry. Micropatterned grooves restrict lateral spreading and promote uniaxial alignment, thereby organizing focal adhesions, actin stress fibers, and intracellular force transmission along a preferred direction. In muscular and endothelial cells^117^, such anisotropic guidance facilitates maturation by enhancing integrin-mediated FAK signaling and associated pathways, including MAPK signaling, resulting in improved sarcomeric organization, electrical coupling such as connexin 43 expression, and spontaneous contractile activity^118,119^. By bioprinting grooves or aligned filament patterns at defined angles, biofabrication can generate anisotropic cardiac tissues that direct electrical propagation and partially reproduce the left ventricle’s helical organization^28^.

Beyond local matrix properties, native tissues are also regulated by dynamic external inputs that arise from their integration with systemic environments. In vivo, blood flow continuously delivers oxygen and nutrients while simultaneously imposing shear stress on endothelial surfaces. These mechanical and transport cues are difficult to reproduce in conventional static culture, but biofabrication enables tissue coupling with perfusion systems and bioreactors that provide controlled external stimulation. For example, Homan et al. developed a 3D-printed millifluidic chip in which kidney organoids were first anchored to a gelatin–fibrin coating through integrin-mediated adhesion and then exposed to a defined medium flow. Compared with static or low-flow conditions, the high-flow culture enhanced vascularization and promoted the maturation of the tubular and glomerular compartments^120^. This response is consistent with established endothelial mechanotransduction pathways, in which molecules such as PECAM-1, VE-cadherin, and Notch1 sense shear stress, leading to downstream signaling that promotes endothelial alignment, junction stabilization, and controlled proliferation under flow^121^ (Fig. 4b). In addition to externally applied flow, engineered tissues can also experience internally generated mechanical loading arising from cell-mediated contraction. This principle is well illustrated in EHTs, in which cardiomyocytes and cardiac fibroblasts are embedded within a matrix and cultured between two anchors^122^ (Fig. 4c). As the tissue contracts, the anchored geometry imposes uniaxial tension and cyclic mechanical resistance, thereby providing physiologically relevant loading cues that promote tissue alignment and maturation. These mechanical inputs are transduced through cytoskeletal tension, integrin-associated signaling, and stretch-activated ion channels such as Piezo and TRP family members, which couple mechanical deformation to calcium influx and downstream signaling^123^. Together with YAP/TAZ-associated mechanoregulation, these pathways contribute to EHTs’ structural and functional maturation. Electrical cues represent a distinct class of dynamic extracellular inputs that can be integrated into biofabricated tissues to regulate collective tissue behavior over time. Unlike static biochemical or structural cujes, external electrical stimulation (E-stim) directly perturbs membrane potential, ion transport, and excitation dynamics, thereby enabling biofabricated systems to reproduce aspects of electrophysiological communication that are otherwise difficult to capture in conventional culture^124^. This role is particularly evident in electrically excitable tissues such as the heart, where physiological function depends on coordinated propagation of electrical activity across multicellular networks. In induced pluripotent stem cell (iPSC)-derived cardiac tissues, E-stim promotes maturation by driving repeated excitation–contraction cycles that reinforce Ca²⁺ handling, action-potential propagation, gap-junctional coupling, sarcomere assembly, and contractile force generation^125^. These functional improvements are accompanied by increased energetic demand, which can further stimulate mitochondrial biogenesis and oxidative metabolic maturation. Importantly, the influence of electrical cues is not restricted to excitable tissues. E-stim can also modulate indirectly responsive or nominally non-excitable cell populations^126^ by altering secretory activity, intracellular signaling, and multicellular interactions. In neurons and endothelial cells, for example, electrical inputs have been associated with changes in growth factor release and activation of signaling pathways such as PI3K–AKT, suggesting that electrical stimulation can reshape the extracellular signaling milieu as well as cell-intrinsic behavior^127,128^. These effects are particularly relevant in multicompartment biofabricated models, where one electrically responsive population may in turn regulate the maturation, migration, or function of neighboring cells through secondary paracrine or matrix-mediated mechanisms^129^.

These examples show how biofabrication controls cellular behavior by engineering ECM properties and the extracellular microenvironment. Matrix composition, ligand density, stiffness, degradability, porosity, and architecture can be tuned to control adhesion, mechanotransduction, migration, and lineage specification. In parallel, scaffolds can be integrated through biofabrication strategies as a mechanical support or a perfusable channel and electrode to guide tissue alignment, impose physiological loading, deliver flow, or provide E-stim. However, ECM design involves inherent tradeoffs because the same material parameters regulating cell signaling also determine printability, structural fidelity, diffusion, remodeling, and long-term tissue viability^130^. For example, increasing matrix stiffness may enhance mechanotransductive signaling and shape retention, but it can also impair cell migration, viability, and tissue remodeling. Similarly, highly permissive matrices may support cell spreading and self-organization but lack the mechanical stability required for reproducible biofabrication. Therefore, engineering functional tissues requires not only selecting an optimal ECM condition at the time of fabrication but also considering the ideal evolution of matrix properties during culture. Recent advances in dynamically tunable hydrogels, degradable matrices, and stimuli-responsive biomaterials provide opportunities to adapt the extracellular environment over time, matching the changing requirements of cell survival, assembly, maturation, and function. In this way, biofabrication can be used to design temporally coordinated cell–ECM communication rather than simply providing an initial scaffold for tissue formation.

Cell–extracellular communication also plays a crucial role in tumor progression and the regulation of tumor–stroma communication. As discussed in Section “Current Approaches to Recreating Biological Crosstalk in a Pathological Context”, cells sense alterations in the biochemical composition and mechanical properties of the matrix through cell-surface receptors, including integrins, CD44, and discoidin domain receptors, which mediate adhesion to matrix components such as collagens, fibronectin, and HA. However, control over these properties is limited in conventional culture platforms. 2D culture and Matrigel-based organoid models fail to capture their in vivo diversity, while animal models do not permit independent control over individual matrix parameters despite their physiological complexity. This limitation is particularly consequential in the TME, where progressive ECM accumulation and remodeling generate abnormal matrix changes that not only result from cellular activity within the TME but also reciprocally drive cancer cell drug resistance and invasiveness. This positions the ECM as a clinically relevant variable in cancer treatment rather than merely a passive structural scaffold. Recent studies^131,132^ have therefore sought to recapitulate tissue-specific ECM composition, dynamically control its physical properties to examine the effects on tumor cell behavior, or engineer defined ECM alignment to investigate its role in immune cell infiltration.

**Fig. 5:**
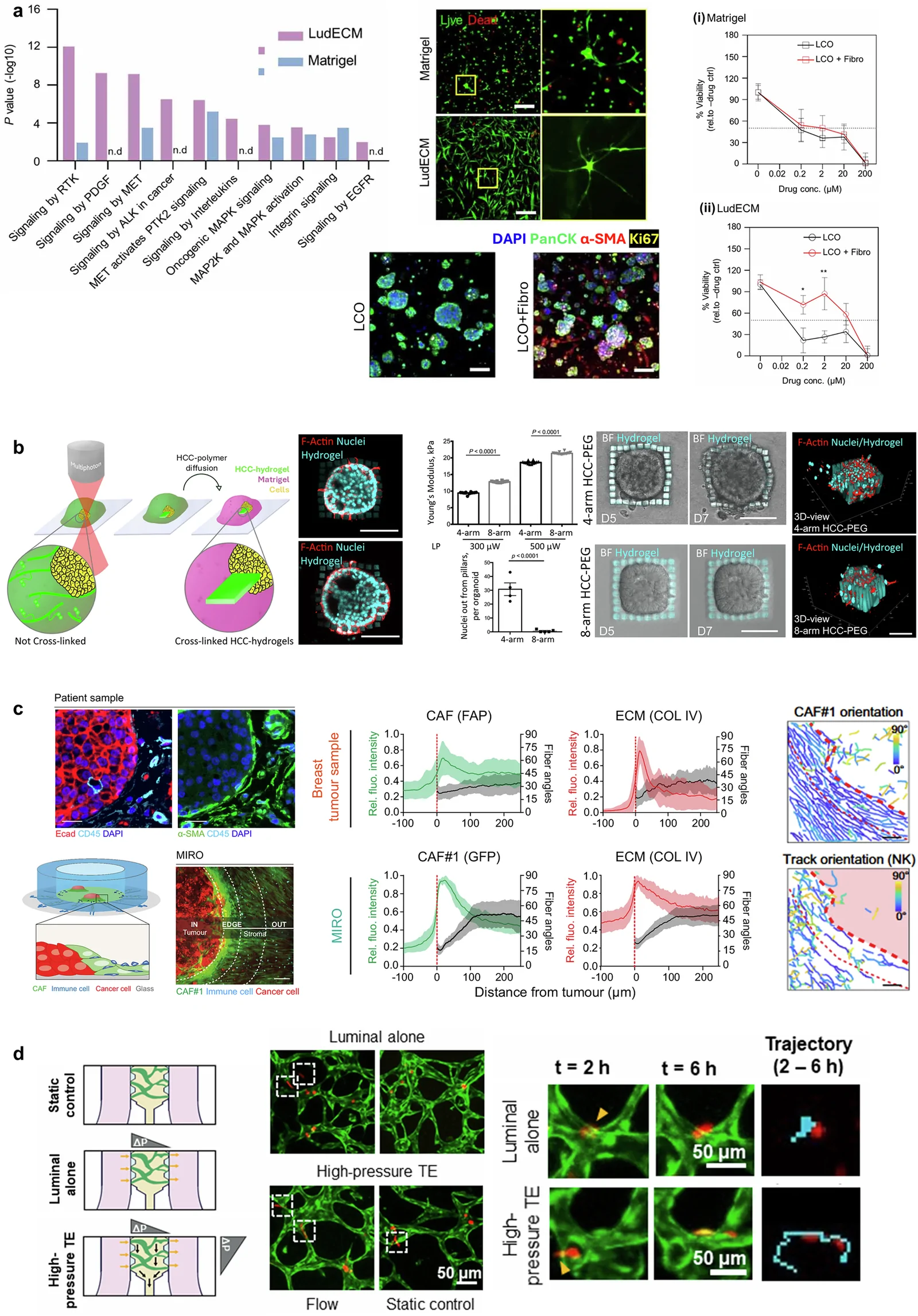
Pathological examples in ECM-mediated regulation of biochemical, biophysical, and external cues. **a** Tissue-specific niche within dECM hydrogel supports ECM-cell interactions, fibroblast morphology, and resistance to EGFR-TKIs. Reproduced with permission^136^, Copyright 2023, IOP Publishing Ltd. **b** Bioprinted pillar arrays generated with two-photon-mediated dynamic crosslinking revealed how physical confinement regulates cancer cell migration. Reproduced with permission^143^, Copyright 2023, Springer Nature. **c** Aligned fibroblasts and ECM fibers surrounding the tumor spheroid guide the migratory track of NK cells. Reproduced with permission^144^, Copyright 2025, Springer Nature. **d** Control of luminal and trans-endothelial (TE) flow with pressure gradients affects the extravasation behavior and invasion trajectory of tumor cells. Reproduced with permission^148^, Copyright 2021, Elsevier Ltd.

In this context, the use of dECM bioinks has emerged as an effective strategy^131^, as they provide physiologically relevant matrix environments for tumor cells across diverse cancer models^132–135^. To approximate a tissue-specific ECM niche, Choi et al. developed a lung-derived dECM (LudECM) hydrogel containing lung-specific ECM components, including collagen VI, fibronectin, and laminin-521^136^ (Fig. 5a). Compared to Matrigel, LudECM, with its diverse matrisome composition, supported broader ECM–cell interactions and enriched cancer-related signaling pathways, including RTK, ALK, and MAPK signaling associated with tumor progression and aggressiveness. Functionally, LudECM induced distinct fibroblast morphology and enhanced collagen deposition and, under co-culture conditions with lung PDOs and idiopathic pulmonary fibroblasts, markedly increased resistance to epidermal growth factor receptor tyrosine kinase inhibitors (EGFR-TKIs). These findings indicate that LudECM can better recapitulate patient-specific TME features associated with underlying fibrotic lung disease than Matrigel.

While dECM-based matrices provide tissue-specific biochemical cues, the precise control of their composition and mechanical properties is inherently difficult; consequently, the use of synthetic hydrogels with tunable biochemical and biomechanical properties has emerged as an alternative strategy to isolate these effects under precisely controllable conditions^110,137–141^. For example, to dissect stiffness-driven chemoresistance, LeSavage et al. engineered an HA–elastin-like protein hydrogel with independently tunable biochemical and mechanical stiffness^142^. Through interchangeable RGD/RDG motifs, HA/PEG polymers, and adjustable crosslinking density, they demonstrated that cancer cells sense matrix stiffness through the HA–CD44 signaling axis, which induces a phenotypic shift characterized by the upregulation of ATP-binding cassette (ABC) drug efflux transporters, ultimately promoting chemoresistance. Biofabrication also enables researchers to dynamically investigate how localized geometric confinement regulates tumor cell migration, a process that is difficult to study using static bulk hydrogels. Urciuolo et al. employed a hydrogel-in-hydrogel live bioprinting strategy that combined photosensitive hydrogels with two-photon–mediated crosslinking, thereby enabling spatiotemporal control over local hydrogel geometry, stiffness, and position^143^ (Fig. 5b). Using bioprinted pillar arrays surrounding tumor organoids, they imposed defined physical confinement and observed a sequential migration process in which cellular projections extended first, followed by translocation of the cell body through the inter-pillar spaces. The modulation of pillar spacing and mechanical properties further showed that the degree of physical confinement regulates the extent of cancer cell migration.

At tumor–tissue boundaries, spatial ECM architecture—including fiber alignment, structural anisotropy, and boundary organization—can guide cell migration, such as cancer cell invasion and immune cell infiltration. To reconstruct this tissue boundary architecture, Perucca et al. created an open fluidic device to model the spatial organization of the breast tumor–stroma boundary and its interaction with immune cells^144^ (Fig. 5c). Specifically, they positioned cancer spheroids on a CAF monolayer while using an interposed channel to define a migratory path for immune cells. Importantly, this study recapitulated aligned ECM fibers and fibroblast orientation along the tumor boundary, revealing that this stromal architecture guides immune cell migration and promotes immune exclusion. These findings illustrate how controlling ECM architecture at tissue boundaries can shape immune cell trafficking and tumor–stroma communication.

Beyond matrix composition and mechanics, flow-derived dynamic extracellular environments are also essential components of cell–extracellular communication in the TME. Dynamic flow through luminal, trans-endothelial, and interstitial regions exposes tumor and stromal cells to biomechanical stimuli, including fluid shear stress and pressure-driven forces, while simultaneously regulating the transport and spatial distribution of nutrients, oxygen, and soluble factors. To recapitulate and control flow conditions in vitro, researchers have employed fluidic platforms that generate continuous luminal flow through perfusable vascular channels while simultaneously imposing IF across cancer cells^145–147^. To reconstruct these cues in a controllable manner, Hajal et al. designed a microfluidic vascular platform that generates perfusable microvascular networks and applied defined hydrostatic pressure gradients to independently impose luminal and trans-endothelial flows across endothelial vessels embedded in a 3D matrix^148^ (Fig. 5d). This biofabrication-enabled fluidic setup allowed them to decouple distinct flow modes that are usually intertwined in vivo. Using this approach, they showed that luminal flow increases tumor cell extravasation by enhancing intravascular migration speed, whereas trans-endothelial flow accelerates transmigration across the endothelium and promotes interstitial invasion. In parallel, controllable fluidic platforms have allowed mechanistic studies of flow-mediated signaling, revealing that IF potentiates exogenous TGF-β-induced Smad signaling and EMT-associated responses^149^, while fluid shear stress enhances the expression of cancer stemness–related markers such as CD44, Nanog, and Oct4^150^. Such platforms have also enabled the systematic dissection of the process by which flow-driven transport and pressure dynamics regulate lymphangiogenesis^148^, immune cell trafficking^151^, and ECM remodeling^152^ in the TME.

Within the TME, ECM composition, stiffness, and architecture are tightly interconnected, such that manipulating any one parameter in isolation may compromise the physiological relevance of engineered models. The crosstalk between these cues and tumor cell behavior is similarly complex; for example, increasing matrix stiffness can enhance mechanotransduction-driven chemoresistance but simultaneously restrict the migratory capacity needed for invasion. Therefore, designing biofabricated tumor models involves simultaneously optimizing ECM composition and mechanical properties and benchmarking the selected parameter ranges against patient-derived tissue data.

### Spatial regulation of heterogeneous tissues enabling intertissue communication

Reconstructing intertissue communication in vitro is essential because many physiological and pathological functions arise not from isolated cell states but from signal exchange across anatomically distinct yet functionally coupled tissues. Such communication is mediated by multiple modes of indirect cell–cell signaling, including paracrine signaling by morphogens, growth factors, and cytokines, synaptic signaling by neurotransmitters, and endocrine signaling by circulating hormones. These signals are inherently spatial; thus, their amplitude, persistence, and biological consequences depend on the relative positions of the signaling and responding tissues, the geometry of their interface, the distance over which ligands or projections must travel, and the timing of their contact. For this reason, deterministic spatiotemporal regulation is not merely a manufacturing advantage but a mechanistic requirement for reliably modeling intertissue communication^153,154^.

**Fig. 6:**
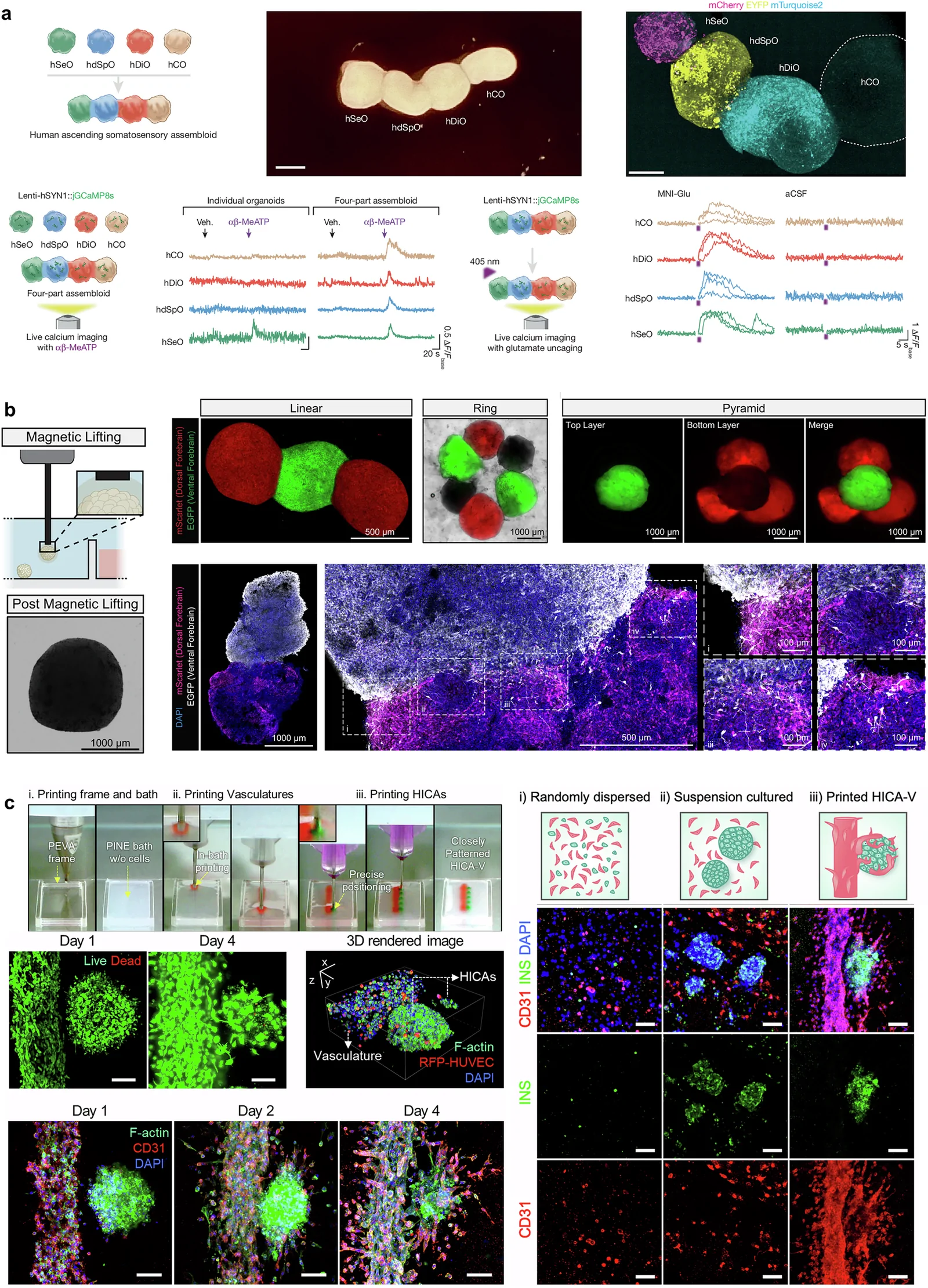
Physiological examples in spatial regulation of heterogeneous tissues enabling intertissue communication. **a** Human ascending somatosensory assembloid model showed functional connectivity and stimulus responsiveness. Reproduced with permission^38^, Copyright 2025, Springer Nature. **b** Magnetic bioprinting enabled positioning of assembloids in linear, ring, and pyramid structures. Reproduced with permission^46^, Copyright 2023, Springer Nature. **c** Imposing guidance of tissue geometry, boundary shape, and islet-vasculature spacing through bioprinting enhanced structural features and islet functions. Reproduced with permission^98^, Copyright 2025, Springer Nature.

Co-differentiation within a single organoid through temporally coordinated developmental cues was proposed as an initial strategy for generating interacting tissues^155^. However, because distinct tissue identities require different morphogen conditions and maturation timelines, this approach offers limited control over spatial arrangement^38^. This limitation has motivated the widespread adoption of assembloids, in which regionally specified organoids or specialized cells are generated separately and then fused^35,156^. Recent assembloid systems have extended this logic further by integrating a greater number of tissues into ordered pathways^37^. For example, in the human assembloid model of the ascending neural sensory pathway, four separately patterned organoids representing cortex, diencephalon, spinal cord, and sensory ganglion-like tissues were assembled to reconstruct the spinothalamic axis^38^ (Fig. 6a). The resulting system displayed functional connectivity and stimulus responsiveness, thereby enabling the study of polysynaptic sensory transmission in a human in vitro context that is otherwise experimentally inaccessible.

However, as discussed in Section “Bioassembly”, conventional assembloid formation still relies largely on manual fusion, which affords only coarse control over inter-organoid distance, sequence, orientation, interface geometry, and higher-order 3D arrangement^157^. Because these spatial parameters directly govern ligand diffusion, tissue connectivity, and signaling exposure, such limited control constrains the reproducibility and mechanistic interrogation of intertissue communication. To address this limitation, Roth et al. developed a magnetic pick-and-place bioprinting platform in which organoids are coated with iron-oxide nanoparticle-laden cellulose nanofiber hydrogel, lifted by an electromagnet-modified printer, and deposited into a self-healing support bath with control in the X, Y, and Z dimensions^46^ (Fig. 6b). Crucially, the method preserved neural organoid cytoarchitecture better than aspiration-assisted bioprinting and enabled the construction of non-trivial configurations, including linear, ring-like, and multilayered assembloids. In dorsal–ventral forebrain assembloids, this controlled positioning supported robust interneuron migration after fusion.

Biofabrication becomes even more important when the goal is not simply tissue assembly but the encoding of interface geometry, boundary shape, and intertissue spacing as functional regulators of crosstalk. Kim et al. fabricated an islet-specific niche by using bioprinting-based geometrical guidance to recreate the spatial arrangement of islet peripheries and tubular vasculature^98^ (Fig. 6c). This engineered geometry and controlled distance between two tissues promoted coordinated islet–vascular interactions, enhanced junctional features, and improved glucose-stimulated insulin secretion compared with non-patterned controls.

Taken together, these examples highlight biofabrication’s value as a strategy for assembling mature or regionally specified tissue units into higher-order systems with controlled spatial organization. In contrast to conventional co-culture or manual fusion approaches, biofabrication defines intertissue distance, orientation, interface geometry, and three-dimensional organization as controllable experimental variables, enabling intertissue communication to be studied with greater reproducibility and mechanistic precision. In addition, multi-material biofabrication enables the integration of distinct tissue compartments, ECM compositions, and cellular architectures within a single construct, including spatially interpenetrating elements such as vascular networks, neural projections, or capillary-like interfaces. However, as the number and complexity of tissue components increase, maintaining the intended spatial organization becomes increasingly difficult. Different cell types and tissue modules may exhibit distinct proliferation rates, contractile behaviors, remodeling capacities, and maturation timelines, causing the initially fabricated geometry to drift over time. These dynamic changes can alter diffusion distances, interface stability, and signaling exposure, thus complicating the interpretation of intertissue communication. Therefore, future biofabrication strategies for modeling intertissue crosstalk should account not only for the precision of initial tissue placement, but also for the dynamic evolution of multicompartment architectures during culture. This will require coordinated control of tissue interfaces, matrix remodeling, and spatiotemporal signaling across compartments. Integrating programmable tissue positioning with adaptive biomaterials, and longitudinal monitoring may enable more stable and physiologically relevant in vitro models of intertissue communication.

**Fig. 7:**
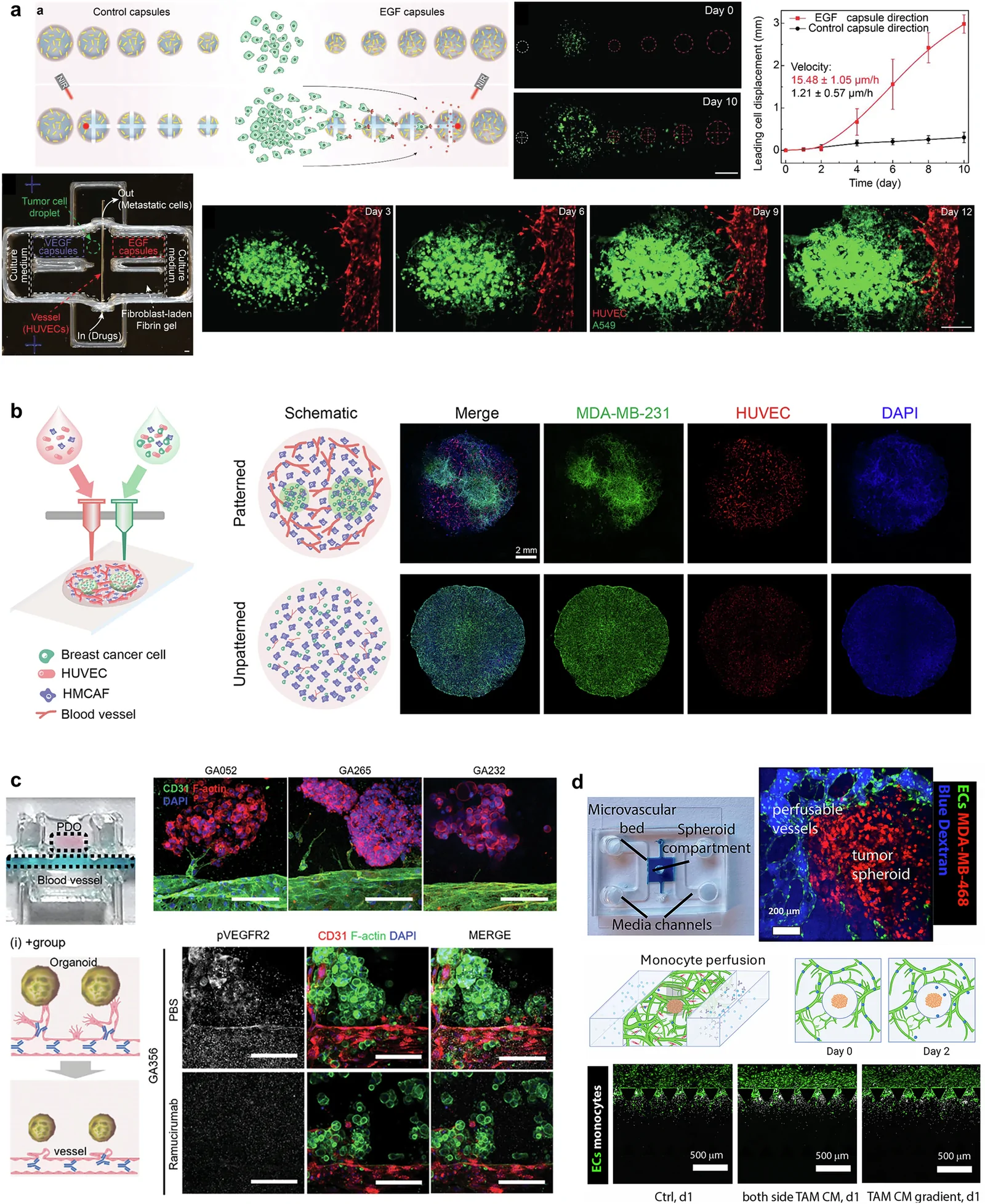
Pathological examples in spatial regulation of heterogeneous tissues enabling intertissue communication. **a** Spatiotemporal control of the release of EGF and VEGF influences the migration and angiogenesis of endothelial cells. Reproduced with permission^158^, Copyright 2019, Wiley–VCH GmbH. **b** The compartmentalized tumor model showed spatially confined angiogenesis and ECM remodeling. Reproduced with permission^166^, Copyright 2024, Elsevier Ltd. **c** Precise positioning of vasculature and patient-derived organoids (PDOs) enabled patient-specific angiogenic sprouting and response to anti-angiogenic therapy. Reproduced with permission^169^, Copyright 2023, Wiley–VCH GmbH. **d** Microfluidic-based vascularized tumor model enabled monocyte perfusion and dissection of TAM-driven CCR2-dependent monocyte recruitment. Reproduced with permission^173^, Copyright 2025, Elsevier Ltd.

These spatial principles become even more critical in TMEs, where communication between cancer cells, vasculature, stromal cells, and immune populations is highly distance-dependent and mediated by paracrine signaling. Compartmentalized tissue assembly with defined inter-compartment spacing makes this distance-dependence directly examinable, including its contributions to angiogenesis, invasion, and immune-cell recruitment. To dissect how distinct growth factors coordinate tumor invasion and angiogenesis, Meng et al. used 3D bioprinting to spatially organize lung cancer cells, endothelial cells, fibroblast-laden fibrin matrices, and programmable VEGF/endothelial growth factor (EGF)-releasing capsules within a single construct^158^ (Fig. 7a). By temporally releasing signaling molecules locally, they showed that EGF drives tumor cell migration toward vascular regions while VEGF induces new vascular bud formation, thus demonstrating the reciprocal coupling between tumor invasion and angiogenesis. Biofabrication further enabled the imposition of spatially defined oxygen gradients across compartmentalized constructs through selective gas-permeable chamber designs, thereby engineering a controllable transition from hypoxia to normoxia^159,160^. This engineered spatiotemporal oxygen environment recapitulated physiologically relevant metabolic reprogramming, enhanced invasion, patient-specific drug sensitivity, and hypoxia-induced stem-like phenotypes.

With advances in spatial omics technologies, the fact that the tumor core and stromal regions exhibit distinct cellular compositions, spatial architectures, and signaling microenvironments has become increasingly evident^161^. Although the co-culture system of tumor organoids and CAFs reveals that fibroblasts are involved in remodeling the tumor ECM and conferring drug resistance^162^, such models remain limited by uncontrolled cell mixing, which obscures the spatial distribution and directionality of chemokine signaling, constrains region-specific mechanotransductive cues, and increases heterogeneity in pathway activation. To overcome these limitations, bioprinting technology has enabled the spatial organization of tumor–stroma interfaces to be controlled in vitro through the precise placement of cell-laden bioinks at desired locations^159,160,162–165^. For example, Yuan et al. developed a compartmentalized tumor model with a dense cancer core and surrounding stromal region, extruding two distinct cell-laden bioinks with defined architecture^166^ (Fig. 7b). Using this model, researchers recapitulated spatially confined angiogenesis, ECM stiffening, region-specific fibroblast and endothelial gene signatures, identifiable endothelial cell–CAF ligand–receptor interactions, and spatially distinct chemotherapeutic responses. Similarly, spatially controlled tumor models further enable the investigation of immune cells’ interaction with structured tumor–stroma architectures, including their migration along chemokine gradients and engagement in ligand–receptor signaling networks^165,167,168^.

Tumor–vascular communication is also governed by physical proximity, making micrometer-scale control over compartment spacing essential for reconstructing angiogenic signaling in vitro. To test this directly, Kim et al. used bioprinting to position tumor and vascular tissues at defined distances and thereby interrogate how spatial separation regulates VEGF-dependent angiogenic crosstalk^169^ (Fig. 7c). They found that angiogenic sprouting occurred efficiently only when tumor and vascular tissues were within 50 μm of each other and that the resulting angiogenic patterns varied according to patient-specific features retained by the PDOs, enabling personalized evaluation of ramucirumab response. Well-spatially organized vascularized tumor models further allow mechanistic interrogation of metastasis by recapitulating interactions between CTCs and the vascular endothelium during intravasation and extravasation^137,170^. At the same time, they provide a platform to evaluate chimeric antigen receptor T-cell efficacy by modeling T-cell trafficking through perfusable vasculature and trans-endothelial infiltration^112^. To further extend these models beyond the blood vasculature, several studies have incorporated lymphatic vessels into tumor constructs^113^ or introduced tissue-specific stromal components to reconstruct more physiologically relevant vascularized TMEs^171,172^.

Beyond direct tumor–vascular interactions, stromal compartments composed of fibroblasts or tumor-associated macrophages (TAMs) introduce an additional regulatory layer that modulates vascular–tumor–immune communication. To reconstruct this process, Nguyen et al. used a spatially organized vascularized tumor platform in which stromal and vascular compartments were separated while intercompartment signaling was preserved, allowing them to model a TAM-driven recruitment axis under defined chemotactic conditions^173^ (Fig. 7d). Within this platform, stromal TAMs secreted CCL2 and CCL7 to promote CCR2-dependent monocyte recruitment from the perfusable vasculature, and unidirectional chemotaxis assays revealed how endothelial barriers, together with TAM-conditioned gradients, orchestrate the spatial patterning of monocyte migration. This platform further enabled therapeutic interrogation using a multi-specific antibody targeting CSF-1R, CCR2, and TGF-β, which suppressed immunosuppressive TAM phenotypes and abrogated monocyte recruitment, thereby elucidating how macrophage–tumor interactions regulate immune infiltration across vascular interfaces.

However, using several cell components together can be problematic, as the structure can change over time. Cells, especially fibroblasts, remodel their surroundings and exert force, and the construct can contract during culture. As a result, the geometry of each component, or the distance between them, may not adhere to the original design. Moreover, fabricating multiple cell components together often requires a complex process during which the shear stress generated or the long processing time can affect cell viability or phenotype.

## Conclusions and future perspectives

Tissue engineering has advanced rapidly, achieving remarkable success in replicating in vivo tissue functions in vitro, yet the in vitro tissue does not match the complexity and dimensions required for drug discovery or transplantation. The mismatch arises as the tissue size and the amount of crosstalk between cells and the extracellular environment increase, and these overwhelm the single-cell activities studied in 2D. However, recent studies have relied on the ease of the fabrication step or the naïve mimicry of the in vivo structure, with little understanding of the underlying mechanism. This review provided a comprehensive overview of biofabrication strategies, including micromolding, bioprinting, and bioassembly, and the major mechanisms of multiscale biological crosstalk, ranging from intercellular to intertissue communication. Through representative studies, we highlighted how biofabrication-enabled spatiotemporal regulation can reconstruct spatially encoded biological crosstalk in vitro. First, morphological regulation of cellular arrangements allows precise patterning of epithelial architectures, organoid structural uniformity, and vascular geometries, demonstrating that tissue morphology itself acts as a regulatory parameter governing intercellular interactions. Second, ECM-mediated regulation of biochemical, biophysical, and external cues shows how programmable extracellular matrices can modulate stiffness, composition, and soluble factor gradients to control biochemical signaling and mechanotransduction. Third, the spatial regulation of heterogeneous tissues through compartmentalized assembly and controlled intertissue spacing enables directional and reciprocal signaling between distinct tissue types, facilitating the reconstruction of intertissue communication networks. Together, these advances position biofabrication as not merely a fabrication technology but an emerging integrative spatial programming framework capable of controlling geometry, mechanics, and multicellular architecture to interrogate biological crosstalk under defined conditions.

**Fig. 8:**
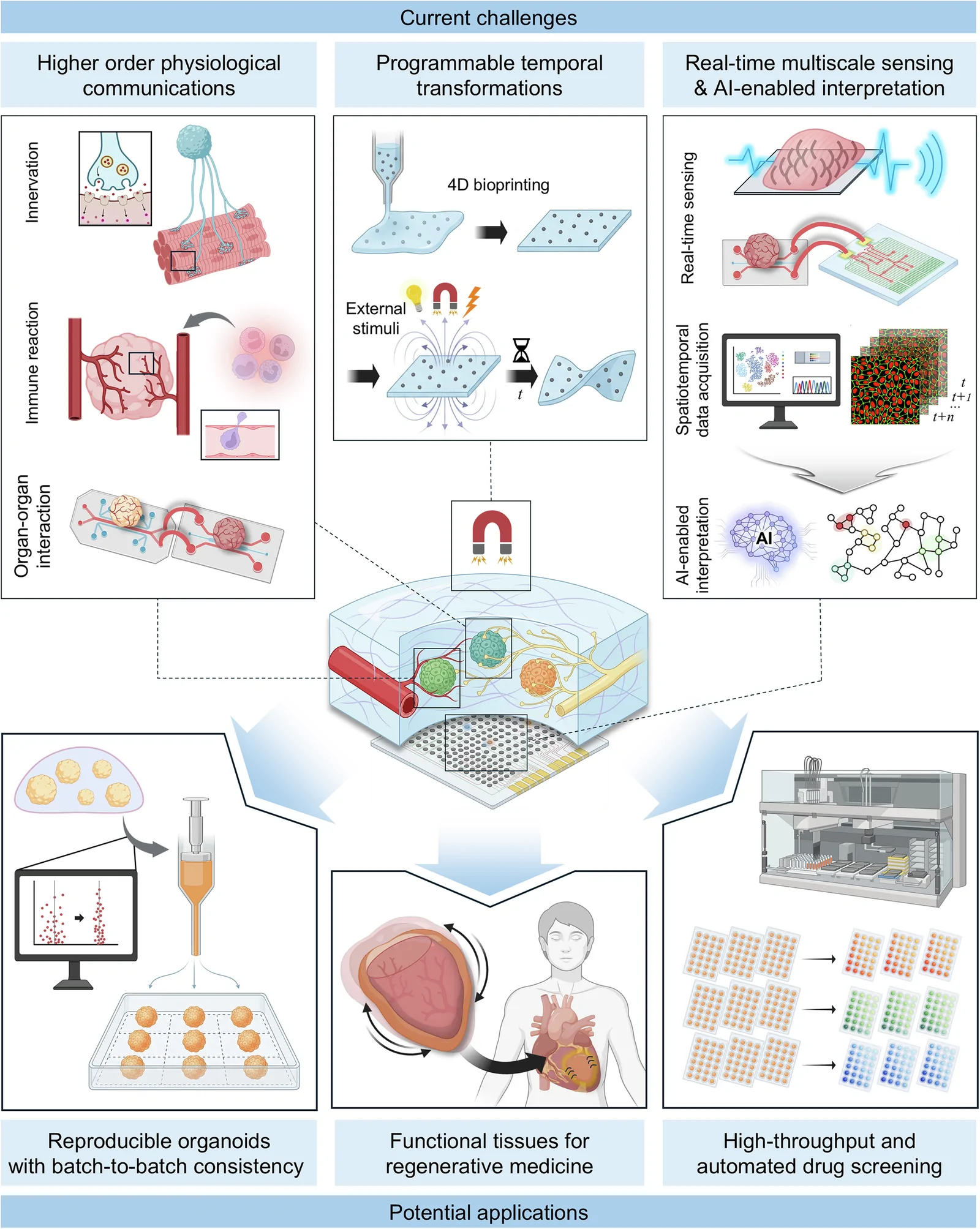
Schematic overview of the current challenges and potential applications.

Although the preceding sections establish the capacity of biofabrication to impose spatial organization on engineered tissues, they also reveal a central unresolved challenge: spatial control at the time of fabrication does not necessarily ensure sustained control over biological crosstalk during tissue maturation. In this section, we discuss three barriers that currently limit the development of spatially programmed tissue systems: multicompartment integration, post-fabrication architectural instability, and the lack of longitudinal, spatially resolved functional readouts (Fig. 8). Here, spatially programmed systems refer to biofabricated in vitro platforms in which the position, spacing, orientation, or interface geometry of multiple cellular or tissue compartments is deliberately prescribed as an experimental variable.

The first barrier is multicompartment integration. Higher-order tissue functions, including vascularization, innervation, immune surveillance^174^, and intertissue communication^175,176^, require the coordinated assembly of parenchymal, vascular, neural, immune, and stromal compartments within a shared construct^7^. The major limitation is no longer the fabrication of each compartment in isolation, but the incompatibility of their biochemical, biophysical, and temporal requirements after integration. Distinct cell populations often require different matrix compositions, stiffness ranges, soluble factor gradients, medium formulations, and maturation timescales. For example, fibroblasts and endothelial cells often proliferate, migrate, and remodel their surrounding matrix more rapidly than co-cultured parenchymal cells, which can cause spatial overgrowth, mechanical distortion, or premature remodeling before the parenchymal compartment reaches functional maturity^177^. Current approaches often address this issue by maturing tissue compartments separately and connecting them only after lineage-specific maturation has been achieved. Although effective in reducing early competition between compartments, this strategy leaves unresolved when compartments should be connected, how compartment maturity should be defined, what constitutes a functional interface, and how the shared culture environment should be regulated after integration.

Addressing this barrier will require a shift from empirical co-culture design toward quantitatively defined compartmental maturation and interface function. Standardized metrics, such as barrier integrity, electrophysiological activity, vascular permeability, or lineage-specific secretory function, could provide rational criteria for staged integration. In parallel, culture environments must become spatially and temporally adaptive. Universal media or fixed mixtures of lineage-specific media are unlikely to support long-term maturation because they cannot accommodate the changing metabolic and developmental demands of each compartment. Artificial intelligence-guided medium optimization may help identify non-intuitive combinations of nutrients and matrix-associated cues that support multiple lineages simultaneously^178^. Biofabrication may further enable compartment-specific regulation through localized perfusion, patterned matrix-bound factors, or spatially confined delivery of extracellular vesicles, thereby allowing distinct tissue regions to receive different maturation signals within a single construct.

The second barrier is post-fabrication architectural instability. Biofabrication can define tissue geometry with high precision at the time of fabrication; however, this architecture is progressively reshaped during culture. Cell proliferation, migration, invasion, ECM degradation, and contraction can alter intercompartment distance, and cellular composition. This architectural drift is particularly problematic when the objective is to determine how spatial parameters regulate biological crosstalk, because the parameter under investigation may no longer correspond to the originally designed geometry.

The conventional response has been to increase matrix stiffness or crosslinking density to preserve the printed structure. However, this approach creates a fundamental trade-off between geometric fidelity and biological permissiveness, because the same material properties that stabilize the construct can suppress cell migration and tissue self-organization. Four-dimensional bioprinting^179,180^ have begun to address post-fabrication shape change, but they still rely on externally imposed triggers, such as temperature, pH, or light, rather than the cell-driven remodeling that dominates long-term maturation. A more effective strategy may therefore be to predict and guide remodeling rather than prevent it. Longitudinal, non-destructive imaging^181^ combined with computational modeling could forecast how initial geometry, matrix mechanics, and cell composition determine remodeling trajectories. These predictions could inform the design of micropattern, geometric anchors, or external stimulation regimes that steer the long-term changes of in vitro tissues^182^. In this framework, cell–extracellular communication becomes a programmable morphogenetic process rather than a source of structural variability.

The third barrier is the lack of longitudinal, spatially resolved functional readouts. Methods that provide detailed molecular information typically require endpoint sampling. These approaches identify the state of a construct at a single time point but cannot track how the same tissue evolves over time. Live imaging and reporter systems provide longitudinal information, but they often monitor a restricted set of predefined events, such as cell migration, reporter activation, or morphological remodeling^183^. This gap becomes increasingly important as biofabricated models incorporate more cell types, larger tissue volumes, and more complex interfaces. Multiscale crosstalk is nonlinear, context-dependent, and temporally dynamic; therefore, endpoint measurements alone are insufficient to determine when a tissue has reached a desired state or when intervention is required.

Closed-loop culture systems provide a promising framework for overcoming this limitation. By integrating live imaging, electrophysiological recording though embedded sensors, and mechanical stress mapping, biofabricated tissues could be monitored continuously during maturation^184^. These data streams could then be interpreted using computational modeling, and further processed in coordinate with omics frameworks to define tissue state and guide timely interventions. Together, these advances would move biofabricated tissue models beyond static structural mimicry toward dynamic, programmable platforms for dissecting multiscale biological crosstalk under defined and controllable conditions.

Despite these opportunities, critical limitations must be acknowledged before spatially programmed systems can be considered to outperform the conventional co-culture, passive organoid fusion, and unpatterned multicellular models they are meant to improve upon. Reproducibility remains a persistent concern: variability in bioink formulations, print fidelity, and post-fabrication maturation can produce functionally heterogeneous constructs even under nominally identical conditions^26^, and batch-to-batch consistency exceeding that of unpatterned culture has rarely been demonstrated. Scalability presents a related gap, as most constructs remain proof-of-concept demonstrations that have not been shown to translate into robust, repeatable platforms^30^. The absence of standardized reporting metrics for spatial resolution, cell viability, architectural accuracy, and functional readouts^14^ compounds both problems by preventing systematic comparison against conventional 2D culture, organoids, or animal models. Validation represents perhaps the most fundamental gap: although many studies report successful spatial patterning, few demonstrate that the resulting constructs are reproducible, biologically stable over time, or measurably superior to their non-patterned counterparts when benchmarked against native tissue organization and function. Biological variability arising from cell source heterogeneity–donor-to-donor differences in primary isolates and line-to-line variation in patient-derived iPSCs–further confounds these comparisons independently of fabrication precision^14^. This absence of systematic benchmarking, together with the lack of standardization and validation, remains a central barrier to the broader adoption and interpretation of spatially programmed systems.

Overall, by enabling deterministic control over tissue architectures, spatially defined cellular interfaces, and programmable extracellular environments, biofabrication-based systems create opportunities to reconstruct these communication networks under experimentally tractable conditions. When combined with rigorous standardization and validation, such spatially structured models could reduce structural variability and enhance experimental reproducibility, and they may contribute to the early-stage evaluation of therapeutic response and toxicity in drug discovery pipelines^171,172^. Moreover, scalable biofabrication strategies that support batch-to-batch consistency^157^, automation compatibility^185^, and integration with high-throughput screening pipelines may accelerate the translation of in vitro models toward industrial and clinical applications. In regenerative medicine, programmable control of cellular positioning and ECM organization further offers a path toward engineering functional tissues with greater structural and dimensional fidelity and improved integration capacity^186,187^. However, achieving vascular integration and immune compatibility at clinically relevant scales will require substantial additional development. By enabling deterministic control of tissue architectures and microenvironmental cues, biofabrication-driven spatiotemporal programming may open new avenues for systematically decoding biological crosstalk and addressing longstanding challenges in disease modeling, drug development, and regenerative medicine.

## Data Availability

No datasets were generated or analysed during the current study.
